# Ginger inhibits the invasion of ovarian cancer cells SKOV3 through CLDN7, CLDN11 and CD274 m6A methylation modifications

**DOI:** 10.1186/s12906-024-04431-3

**Published:** 2024-04-04

**Authors:** Xiaoyu Zhang, Hairong Zhang, Lin Zhu, Lei Xia

**Affiliations:** 1 School of Chinese Medicine, Shandong University of Traditional Chinese Medicine, Jinan, China; 2 Department of Obstetrics and Gynecology, Shandong Provincial Third Hospital, Jinan, 250031 P.R. China; 3 Department of Pathology, Shandong University of Traditional Chinese Medicine, Jinan, 250355 P.R. China

**Keywords:** Ginger, m6A, Ovarian cancer, SKOV3, Plant

## Abstract

**Background:**

Ginger is a common aromatic vegetable with a wide range of functional ingredients and considerable medicinal and nutritional properties. Numerous studies have shown that ginger and its active ingredients have suppressive effects on manifold tumours, including ovarian cancer (OC). However, the molecular mechanism by which ginger inhibits OC is not clear. The aim of this study was to investigate the function and mechanism of ginger in OC.

**Methods:**

The estimation of n6-methyladenosine (m6A) levels was performed using the m6A RNA Methylation Quantification Kit, and RT-qPCR was used to determine the expression of m6A-related genes and proteins. The m6A methylationome was detected by MeRIP-seq, following analysis of the data. Differential methylation of genes was assessed utilizing RT-qPCR and Western Blotting. The effect of ginger on SKOV3 invasion in ovarian cancer cells was investigated using the wound healing assay and transwell assays.

**Results:**

Ginger significantly reduced the m6A level of OC cells SKOV3. The 3’UTR region is the major site of modification for m6A methylation, and its key molecular activities include Cell Adhesion Molecules, according to meRIP-seq results. Moreover, it was observed that Ginger aids significantly in downregulating the CLDN7, CLDN11 mRNA, and protein expression. The results of wound healing assay and transwell assay showed that ginger significantly inhibited the invasion of OC cells SKOV3.

**Conclusions:**

Ginger inhibits ovarian cancer cells’ SKOV3 invasion by regulating m6A methylation through CLDN7, CLDN11, and CD274.

**Supplementary Information:**

The online version contains supplementary material available at 10.1186/s12906-024-04431-3.

## Introduction

Ovarian cancer is among the three major gynaecological cancers, ranking third after cervical cancer and endometrial cancer [1]. As ovarian cancer is insidious at the onset, it is often detected at a late stage, thus delaying treatment. As a result, ovarian cancer is often considered the ‘silent killer’. As a result, ovarian cancer is often referred to as the ‘silent killer’ [2, 3]. Currently, the main treatment for ovarian cancer is surgey, supplemented by chemotherapy, immunotherapy, targeted therapy, and radiotherapy [4, 5]. Although the survival rates for all stages of ovarian cancer have improved with the development of different treatments, new treatments are still being researched.

Ginger is an important spice grown in tropical and subtropical regions [6]. Ginger is used as a spice in everyday life and as a traditional Chinese medicine. According to recent pharmacological studies, ginger has antioxidant, anti-tumour, anti-cough, anti-inflammatory and antiemetic properties [7–9]. Studies have shown that the ethanolic extract of ginger inhibits skin tumourigenesis in mice, and its protective mechanism involves several mechanisms [10]. Park et al. found that 6-gingerol significantly inhibited skin inflammation and suppressed the development of skin papillomas in mice [11]. Population-based epidemiological studies in India have shown that the incidence of many malignancies, including rectal cancer, is lower in South-East Asia than in Western countries because of the high levels of phenolic compounds such as ginger in the daily diet [12].

N6-methyladenosine (m6A) is a ubiquitous intracellular modification in almost all eukaryotic mRNAs and is dynamically regulated by methyltransferases and demethylases to transfer S-adenosine methionine, which in turn is recognized by methyl-binding proteins and regulates life processes. m6A was first identified in poly(A) RNA in 1974 [13, 14]. Its modification abundance is about 1 per 700–800 nucleotides, with a common sequence: RRm6ACH, where R = G/A (G > A) and H = U/A/C (U > A > C), primarily within the G(m6A)C (∼ 70%) or (m6A)C (∼ 30%) sequences. m6A methylation modifications are selectively clustered in mRNA sequences, mainly in the coding sequence (CDS) region (∼ 30%). The m6A methylation modification is selectively clustered in the mRNA sequence, specifically in the CDS region (coding sequence) and the 3’UTR region of the stop codon [15, 16]. With the advancement of bioinformatics techniques and RNA high-throughput sequencing technologies in recent years, m6A-based RNA epigenetic modifications have gradually become a research hotspot in the biological sciences, and have also attracted widespread attention in the field of tumour therapy [17].

These RNA metabolic processes, which are regulated by m6A, play an important role in this range of cellular processes. At the cellular level, it can affect the homeostasis of immune cells, the development of sperm cells, the function of haematopoietic stem cells, the occurrence of cancer cells, the development of nerve cells, and so on. Once the enzyme involved in m6A modification is abnormal, it causes a range of diseases, including tumours, neurological diseases and embryonic developmental delay.

A large number of studies have shown that abnormalities in the m6A modification are involved in the onset and development of tumours such as ovarian cancer, cervical cancer, lung cancer, etc. However, there are often large differences in m6A-related regulatory abnormalities between different tumours, and their downstream targets are also different. Relevant mechanisms include tumour stem cell self-renewal and differentiation, proliferation and apoptosis, invasion and metastasis, drug resistance, immunosuppression and other processes. Therefore, in-depth understanding of the mechanism of m6A modification abnormalities in tumours and targeting key proteins involved in m6A modification are expected to become potential molecular targets for cancer diagnosis and treatment and drug development.

Recently, phytopharmaceuticals have garnered significant attention from researchers due to their potential to modulate disease progression by influencing m6A methylation levels. Shen et al. demonstrated that dihydroartemisinin effectively induced hepatic stellate cell ferroptosis through the regulation of m6A methylation, thereby ameliorating liver fibrosis. Additionally, they found that modulation of m6A methylation attenuated podocyte pyroptosis and mitigated associated injury [18]. Humantenine affected the expression of the human colon cancer cell line (HCT116) through m6A modification [19]. Our previous studies have shown that ginger has some inhibitory effect on SKOV3 cells [20], but whether the mechanism is related to the abnormal level of m6A modification remains unclear. Therefore, this study mainly observed the effect of ginger on m6A modification of SKOV3 cells and searched for its potential targets.

## Result

### Ginger reduced m6A methylation levels

**Fig. 1:**
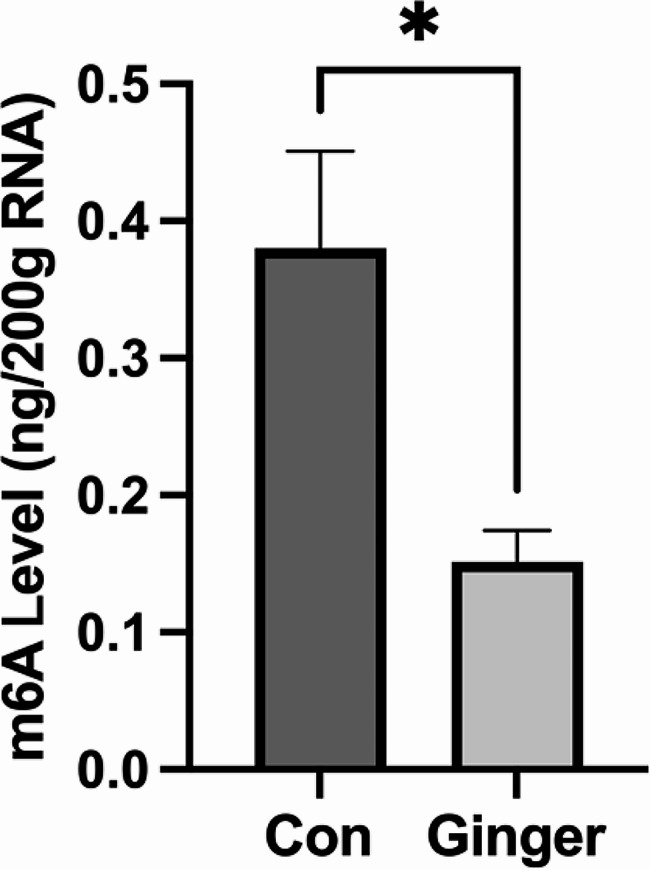
Ginger engendered a reduction in m6A methylation status (*n* = 3). The data were expressed in terms of means (± SEM). *P < 0.05. Con: control group, Ginger: Ginger-treated group

We observed the effect of ginger on the total m6A modification level of SKOV3 cells by colourimetric method and the results showed that ginger can significantly reduce the m6A modification level of SKOV3 cells. It is suggested that the inhibitory effect of ginger on SKOV3 cells may be related to m6A modification (Figure.1).

### m6A-related genes and proteins expression

**Fig. 2:**
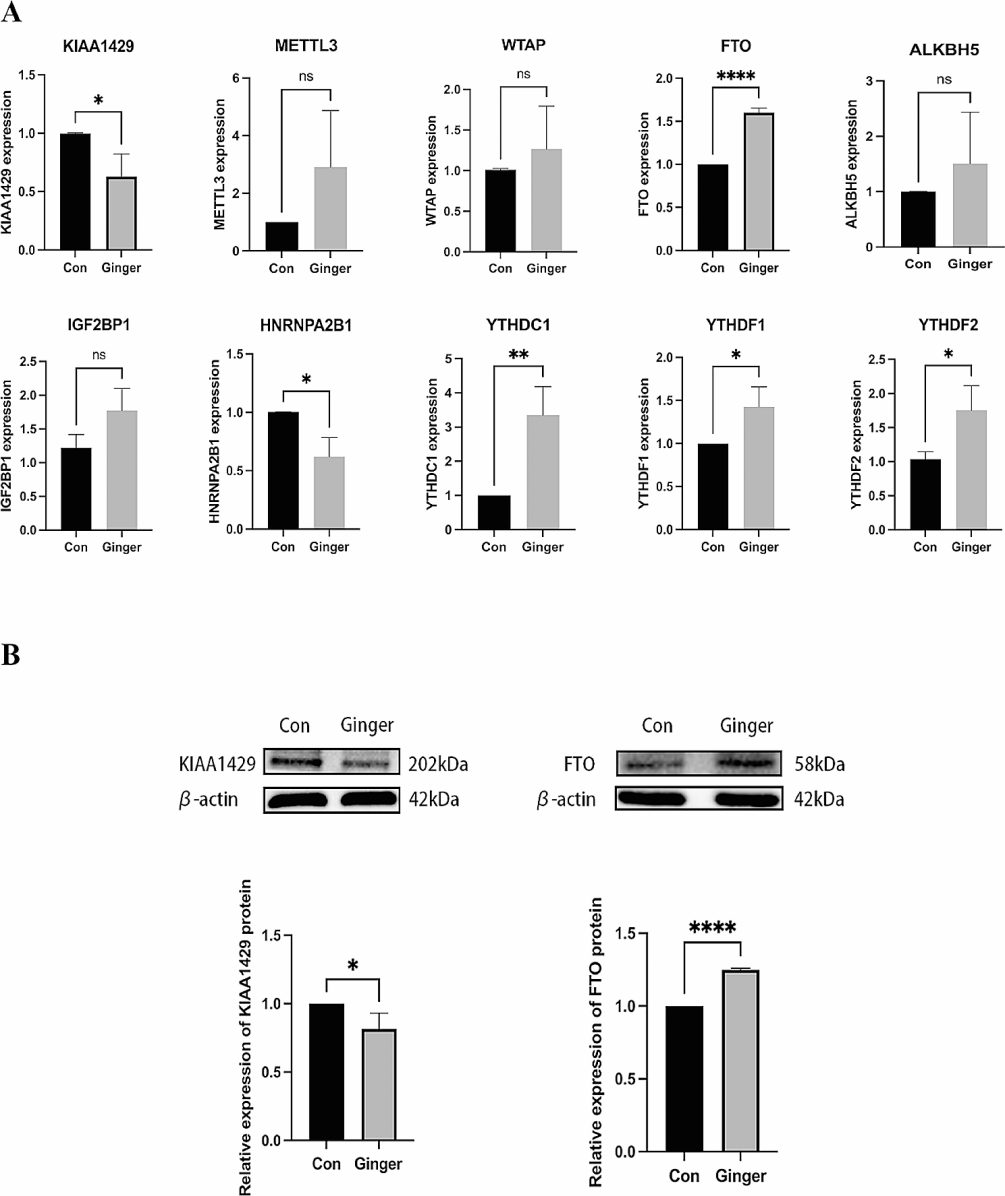
(**A**) Ginger’s impact on the mRNA expression that involves vital genes such as KIAA1429, METTL3, WTAP, FTO, ALKBH5, IGF2BP1, HNRNPA2B1, YTHDC1, YTHDF1, and YTHDF2 was studied. (**B**) The Western blot images of KIAA1429 and FTO serve as representative results. The values are represented in terms of mean (± SEM), having a sample size as *n* = 3. The statistical analysis indicates a non-significant difference (nsp) with *p*-values > 0.05, while the significance level is represented by *=*p* < 0.05, **=*p* < 0.01, and ****=*p* < 0.0001. The study groups are illustrated by Con (control group) and Ginger (ginger-treated group)

We first screened m6A modification-related proteins by RT-PCR, and the results showed that upregulated mRNA expression levels of YTHDC1, YTHDF1, FTO, and YTHDF2 while the expression levels of KIAA1429 and HNRNPA2B1 were found to be downregulated in SKOV3 ovarian cancer cells treated with ginger compared to the control group (*P* < 0.05) (Figure 2A). We further detected the expression of these proteins by Western Blot, and the results showed that FTO increased and KIAA1429 decreased in the ginger treatment group (Figure.2B).

### Transcriptome-wide detection of m6A modification after Ginger Treatment of SKOV3 cells

**Fig. 3:**
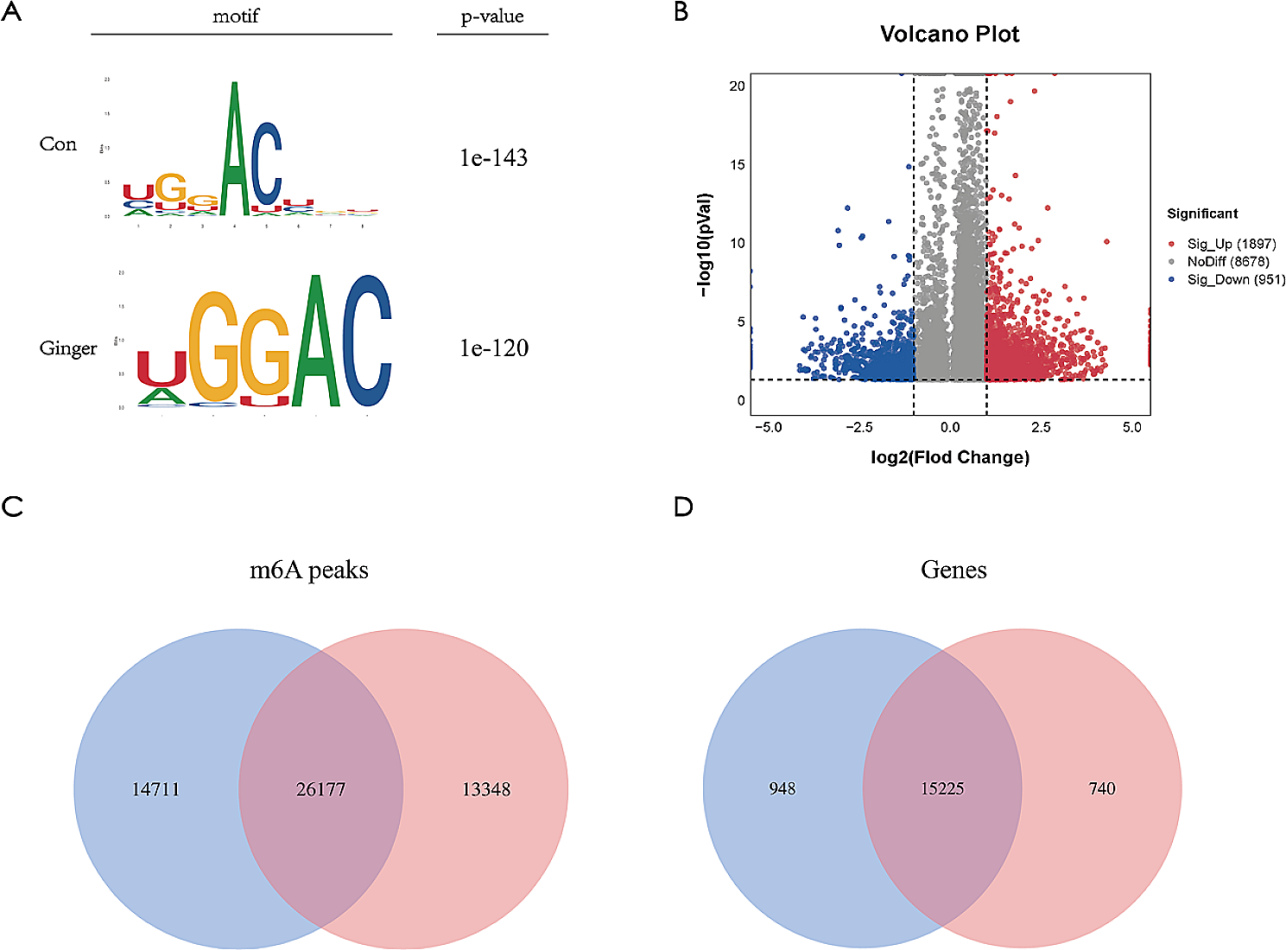
Characteristics pertaining to m6A methylation within the Ovarian cancer cell SKOV3, were examined within the Con and Ginger-treated groups. (**A**) Top consensus motif identified from m6A peaks assessed from the Con and Ginger-treated groups (**B**) Analyzing the proportion of hypermethylated and hypomethylated m6A peaks in the Ginger-treated group in comparison to the Con group. (**C**) Quantification of the number of m6A peaks recognized by m6A-seq in the Con and Ginger-treated groups (**D**) Synopsis of the m6A-altered genes identified using m6A-seq

To investigate the function of ginger in SKOV3 ovarian cancer cells, a MeRIP-seq analysis was performed. We investigated whether the m6A consensus sequence of RRACH (where R, A and H represent purine, m6A and a non-guanine entity, respectively) was seen in the detection of m6A using STREME, as shown in Fig. 3A. Comparisons were made between the m6A peak abundance of the Con and Ginger-treated groups in common m6A-modified genes, revealing 1897 hyper- and 951 hypo-methylated m6A peaks in the Ginger-treated group compared to the Con group (log2|fold change| > 1, while *p* < 0.05; as shown in Fig. 3B). The comparative analysis also revealed 26,177 common m6A modification peaks in both; the Con and Ginger-treated groups, with 13,348 unique m6A peaks in the Ginger-treated group and 14,711 in the Con group (as highlighted in Fig. 3C). Furthermore, we identified 15,225 common m6A-altered genes in Con and Ginger-treated groups, respectively, and 948 and 740 unique m6A-altered genes in the Con and Ginger-treated groups, respectively, as shown in Fig. 3D.

**Fig. 4:**
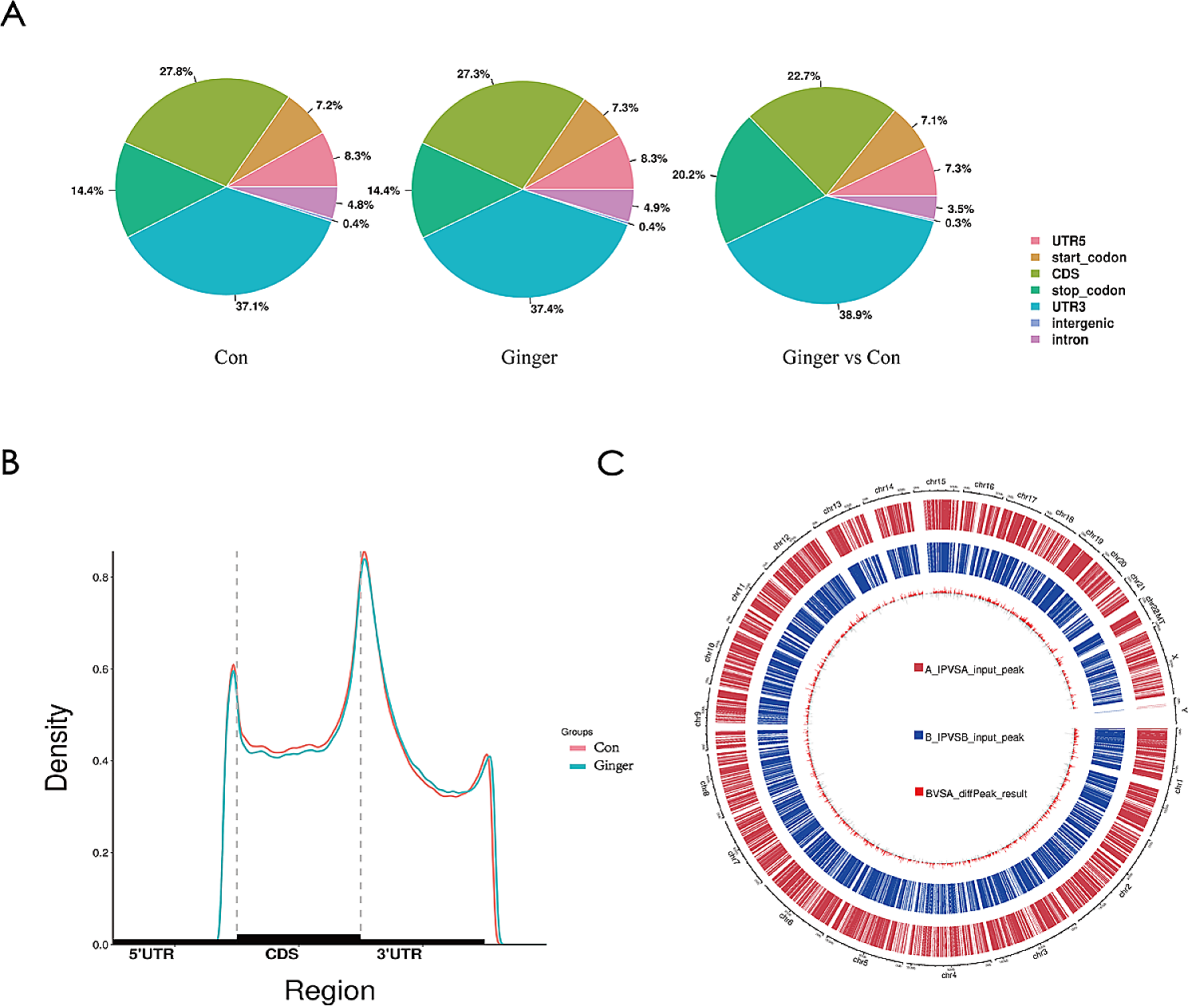
The m6A methylation distribution profiles were examined amongst the two groups. This was accomplished through (**A**) measurement of the percentage and (**B**) accumulation of m6A peaks amongst the six transcript segments; (**C**) and analysis of the distribution of m6A peaks on chromosomes

The m6A methylation distribution patterns were classified into six (06) transcript segments, which include the stop codon (stop C), coding sequence (CDS), the start codon (start C), the 5′ and 3′ un-translated region (5′UTR; 3′UTR), and noncoding sequence. Our analysis revealed the 3′UTR domain as the most vital site of m6A modification, followed by CDS. The maximum peak density was found close to the stop codon, with the density of m6A peaks increasing quickly between the 5′UTR and the start codon but remaining comparatively low throughout the CDS region. In the 3′UTR region, the m6A peak density declined steeply, as shown in Fig. 4.

### Differentially expressed genes are involved in key biological processes and pathways

**Fig. 5:**
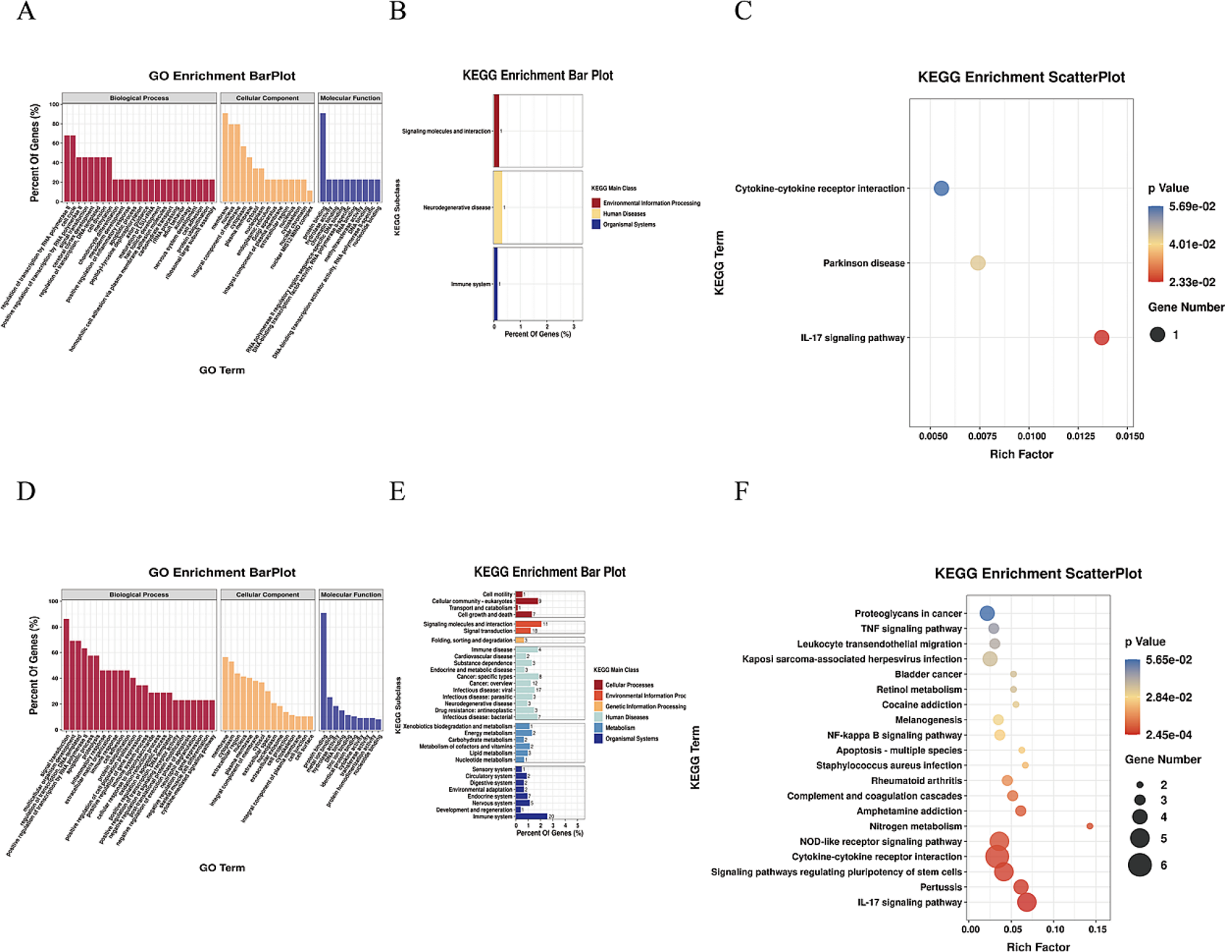
Genes that were differentially regulated among the Ginger-treated and Con groups were analyzed for their biological function and pathway. (**A**) It illustrates the upregulated genes’ GO enrichment analysis portrayed excellent diversity. (**B**) the subclass exploration of KEGG enrichment was done on genes that were upregulated with major differences. (**C**) KEGG pathway enrichment analysis provided insights into the genes that are upregulated and had remarkable diversity. (**D**) The significantly downregulated genes were subjected to GO enrichment exploration. (**E**) KEGG enrichment subclass investigation was conducted on genes that were highly downregulated. (**F**) KEGG pathway enrichments provided valuable information on the significantly downregulated genes

KEGG and GO analyses were performed on the screened DEGs to assess signalling pathways and biological processes, and GO enrichment analyses showed that the up-regulated genes included “RNA polymerase II transcriptional regulation” and “cell cycle” (biological process); “protein binding” (molecular function), and “membrane”, “nucleus”, and " membrane’s integral component” (cellular component). Meanwhile, the down-regulated genes were enriched in “signal transduction”, “multicellular organism development” and “regulation of transcription, “protein binding” (molecular function), DNA-templated” (ontology: biological process); “membrane” and “cytoplasm” (cellular component). These findings are presented in Fig. 5A and D.

Analysis of the KEGG pathway displayed that the upregulated genes associated with the “IL-17 signaling pathway”, were followed by “Parkinson’s diseases” and “Cytokine-cytokine receptor interaction”. The downregulated genes, however, have strong connections to the “IL-17 signaling pathway,” “Pertussis,” and “Signaling pathways modulating stem cells’ pluripotency” (Fig. 5B, C, E, F).

### Differentially methylated genes contributed to significant biological processes and pathways

**Fig. 6:**
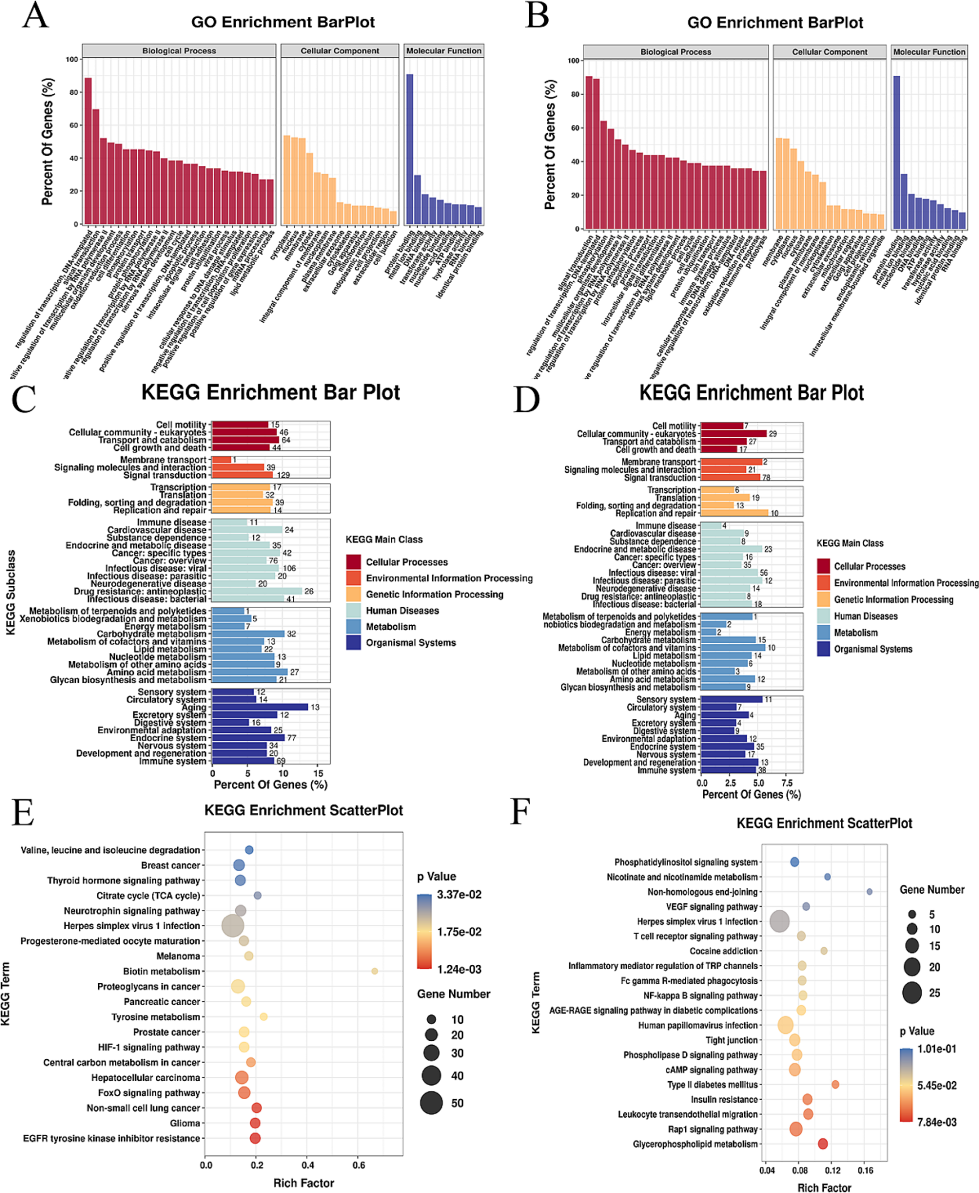
This analysis includes (**A**) an assessment of hypermethylated peaks employing GO enrichment analysis. (**B**) assessment of hypermethylated peaks utilizing the KEGG enrichment subclass. (**C**) Exploration of hypermethylated peaks employing KEGG pathways. (**D**) Exploration of hypomethylated peaks employing GO enrichment analysis. (**E**) Analysis of hypomethylated peaks utilizing KEGG enrichment subclass. (**F**) KEGG pathways involved in the hypomethylated peaks

KEGG pathway and GO enrichment analyses were performed to explore the biological significance of m6A modification in Ginger-treated group. The results of GO analysis showed that the Con and Ginger-treated groups’ hypermethylated and hypomethylated genes were both involved in the following activities and pathways (ontology): nucleus, membrane, and cytoplasm” (cellular component); and " DNA-templated, transcription regulation” and “signal transduction” (biological process); “nucleotide, protein, metal ions, and DNA binding” (molecular function) (Fig. 6A.B). According on KEGG subclass analyses, the following functions and pathways were associated with hypermethylated and hypomethylated genes: “cellular community-eukaryotes, cell motility, growth, and death” (cellular processes); and “signaling molecules, signal interaction, and transduction” (processing of environmental information). Additionally, it was shown that genes that were hypo- and hypermethylated associated to “immune system” (organismal systems) and “immune disease” (human diseases) (displayed in Fig. 6C, D). In terms of KEGG pathway enrichment, hypermethylated genes showed a strong association with “EGFR tyrosine kinase inhibitor resistance” and “glioma” (shown in Fig. 6E). However, there was a strong correlation between the hypomethylated genes and the “Rap1 signaling pathway” and “glycerophospholipid metabolism” (as shown in Fig. 6F).

### RNA-Seq and MeRIP-Seq Data Conjoint analyses

**Fig. 7:**
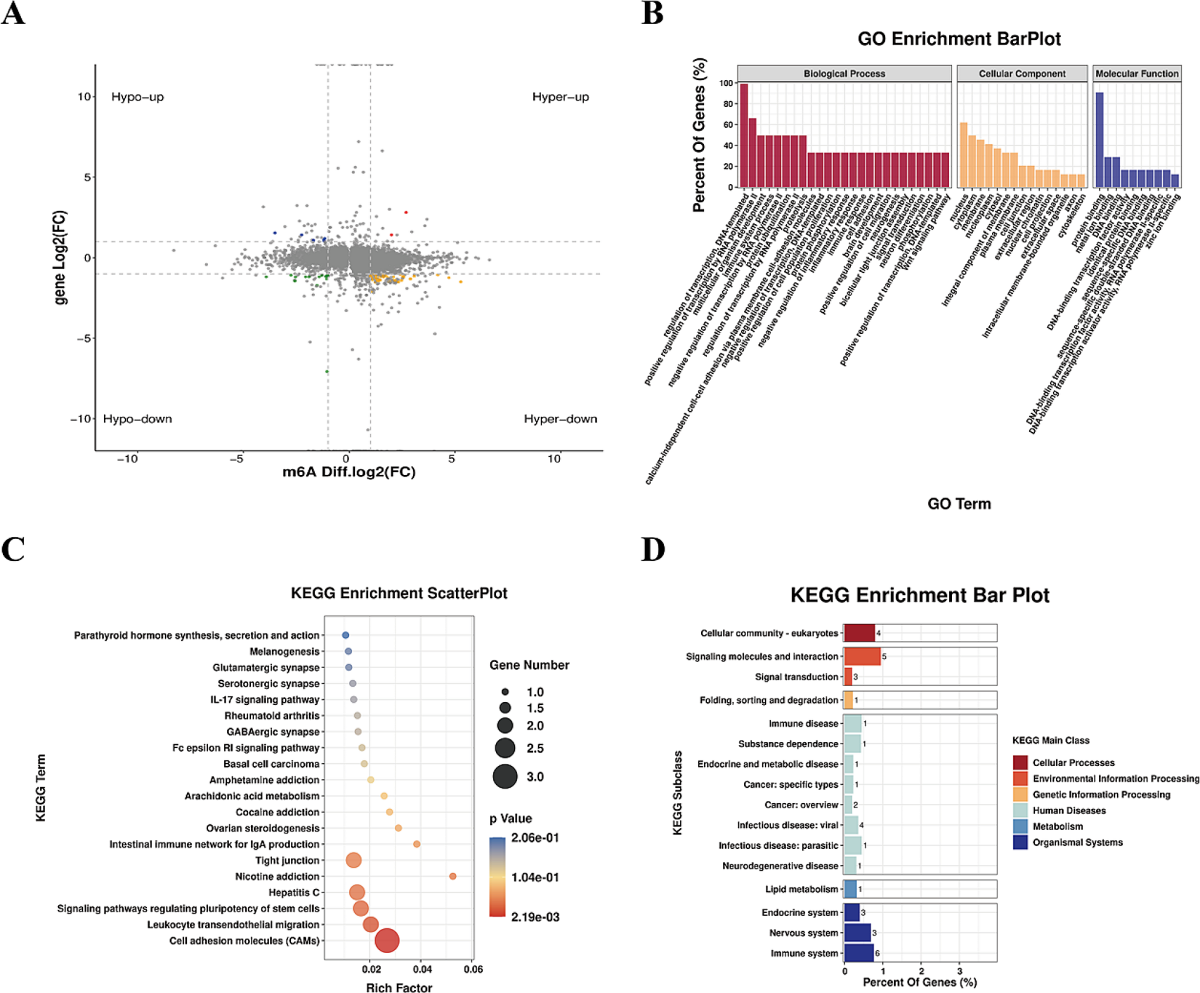
Conjointly analyzing the data from RNA-seq and MeRIP-seq, (**A**) Compared with Con, m6A modification showed significant difference in gene distribution in ginger treatment group. (**B**) Further elucidation is provided through GO enrichment exploration of these genes. (**C**) The KEGG subclass of genes with significant changes in mRNA levels and m6A modification also provides information about their characteristics. Lastly, (**D**) the enrichment analysis of KEGG pathways using differentially expressed genes, both at the m6A modification and mRNA levels, contributes to our comprehension of this phenomenon

Integrating the analytical data of RNA-seq and MeRIP-seq, it was emphasized that a total of 115 differentially methylated and expressed genes (DMEGs) underwent significant changes. These changes consisted of 76 genes that were hypermethylated and downregulated (hyper-down), 7 genes that were both hypermethylated as well as upregulated (hyper-up), 8 genes that were hypomethylated and upregulated (hypo-up) and 24 genes that were hypomethylated and downregulated (hypo-down) (Fig. 7A). Table 1 presents a collection of 20 genes that exhibit differential methylation and expression. Significantly, the GO analysis unveiled that the DEMGs were predominantly linked to biological processes such as “transcription regulation, DNA-templated,” and " RNA polymerase II positive transcription regulation.” In terms of cellular components, they were primarily associated with the “nucleus, cytosol, and membrane.” Furthermore, their molecular functions encompassed “proteins, metal ions, and DNA binding” (Fig. 7B). Likewise, in the KEGG subclass analysis, the DEMGs demonstrated significant involvement in cellular processes such as “cellular community, catabolism, and transport “. Furthermore, they played a crucial role in “signal transduction” within the domain of environmental information processing. Of critical significance, DMEGs were linked to “Cancer: specific types” and “Cancer: overview” concerning human diseases (Fig. 7C). Finally, the analysis of KEGG pathways showed that the DMEGs were predominantly enriched in pathways such as “Signaling pathways regulating the pluripotency of stem cells” “Cell adhesion molecules,” and “Leukocyte transendothelial migration” (Fig. 7D).

**Table 1:** Differentially expressed genes with differentially methylated peaks

Gene	Transcript ID	Chr	Start	End	m6A regulation	FC	*P* value	Gene regulation
SAMD11	ENST00000420190	chr1	924,127	924,377	up	2.06	0.03	down
MATN1-AS1	ENST00000414532	chr1	30,718,928	30,719,228	up	4.18	0.01	down
WNT2B	ENST00000256640	chr1	112,514,849	112,514,999	down	-2.06	0.05	down
NBPF19	ENST00000369227	chr1	149,475,987	149,477,999	up	2.69	0.01	up
CDK18	ENST00000360066	chr1	205,531,637	205,531,737	up	4.71	0.00	down
HNRNPA3P12	ENST00000437410	chr1	53,975,043	53,975,468	up	2.09	0.00	down
PLGLB1	ENST00000409310	chr2	87,002,683	87,002,933	up	2.57	0.04	down
CLDN11	ENST00000064724	chr3	170,433,423	170,433,823	up	2.56	0.00	down
KLHL24	ENST00000242810	chr3	183,679,819	183,679,944	up	2.42	0.00	down
AC111000	ENST00000505646	chr4	69,214,526	69,214,976	up	3.06	0.01	down
LINC00707	ENST00000436383	chr10	6,779,673	6,781,339	down	-2.25	0.01	up
MMP13	ENST00000260302	chr11	102,942,994	102,943,219	down	-2.54	0.00	down
DNAH10OS	ENST00000514254	chr12	123,928,760	123,928,860	up	5.27	0.00	down
ESRRB	ENST00000380887	chr14	76,498,332	76,498,707	up	2.33	0.04	down
ALPK3	ENST00000258888	chr15	84,840,482	84,840,607	down	-3.92	0.01	down
CLUHP3	ENST00000562354	chr16	31,709,876	31,709,976	up	2.89	0.02	down
MEFV	ENST00000219596	chr16	3,249,553	3,249,778	up	2.53	0.01	down
MYADM	ENST00000336967	chr19	53,865,680	53,866,060	down	-2.75	0.00	down
FER1L4	ENST00000611673	chr20	35,564,432	35,564,557	up	2.47	0.01	down
AC073529	ENST00000608176	X	10,860,095	10,860,245	down	-3.52	0.03	up

### Effect of ginger on CLDN7, CLDN11 and CD274 mRNA and protein expression

**Fig. 8:**
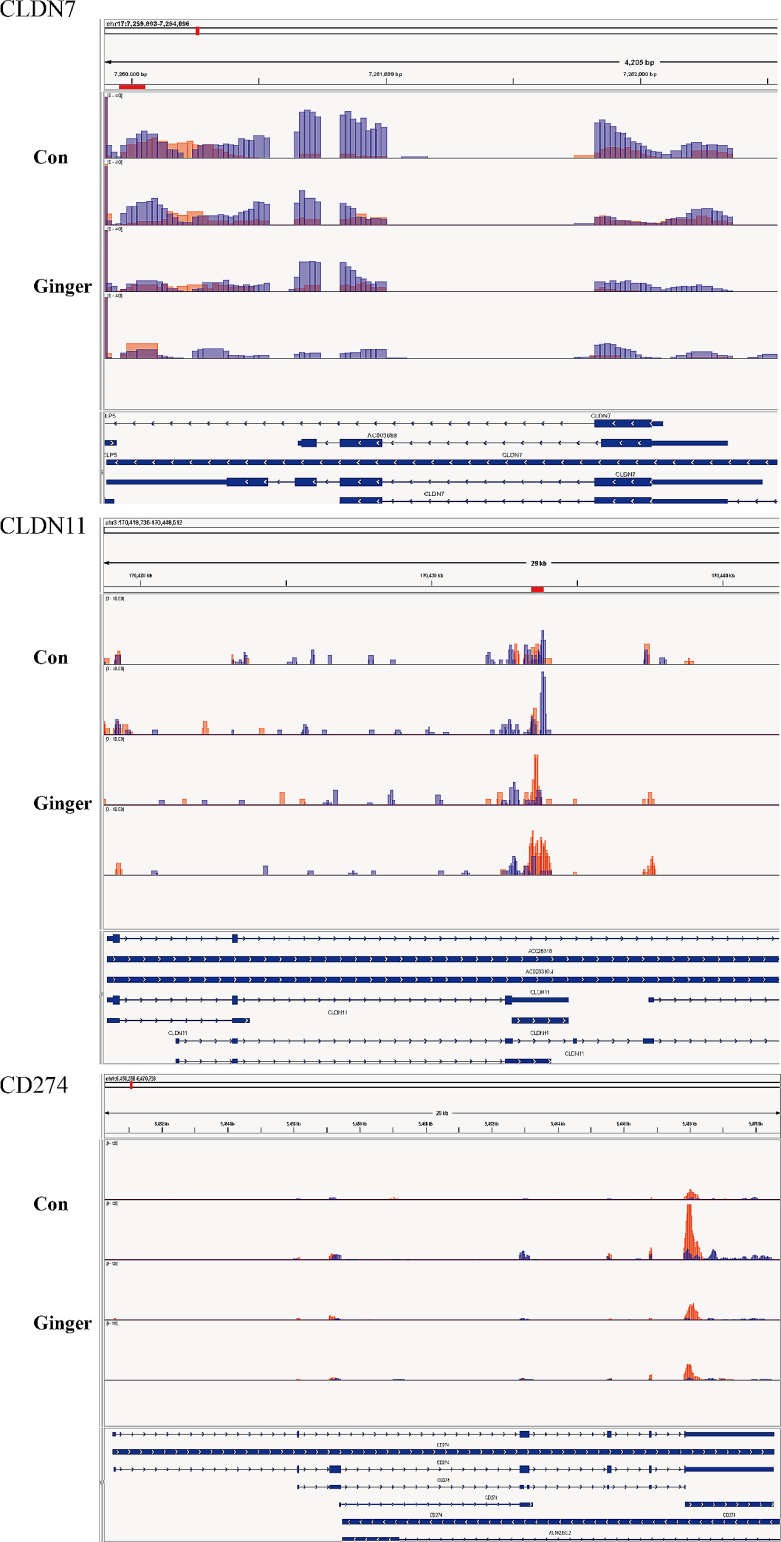
m6A methylation of CAMs-related genes. CLDN7, CLDN11, and CD274, as estimated by IGV

**Fig. 9:**
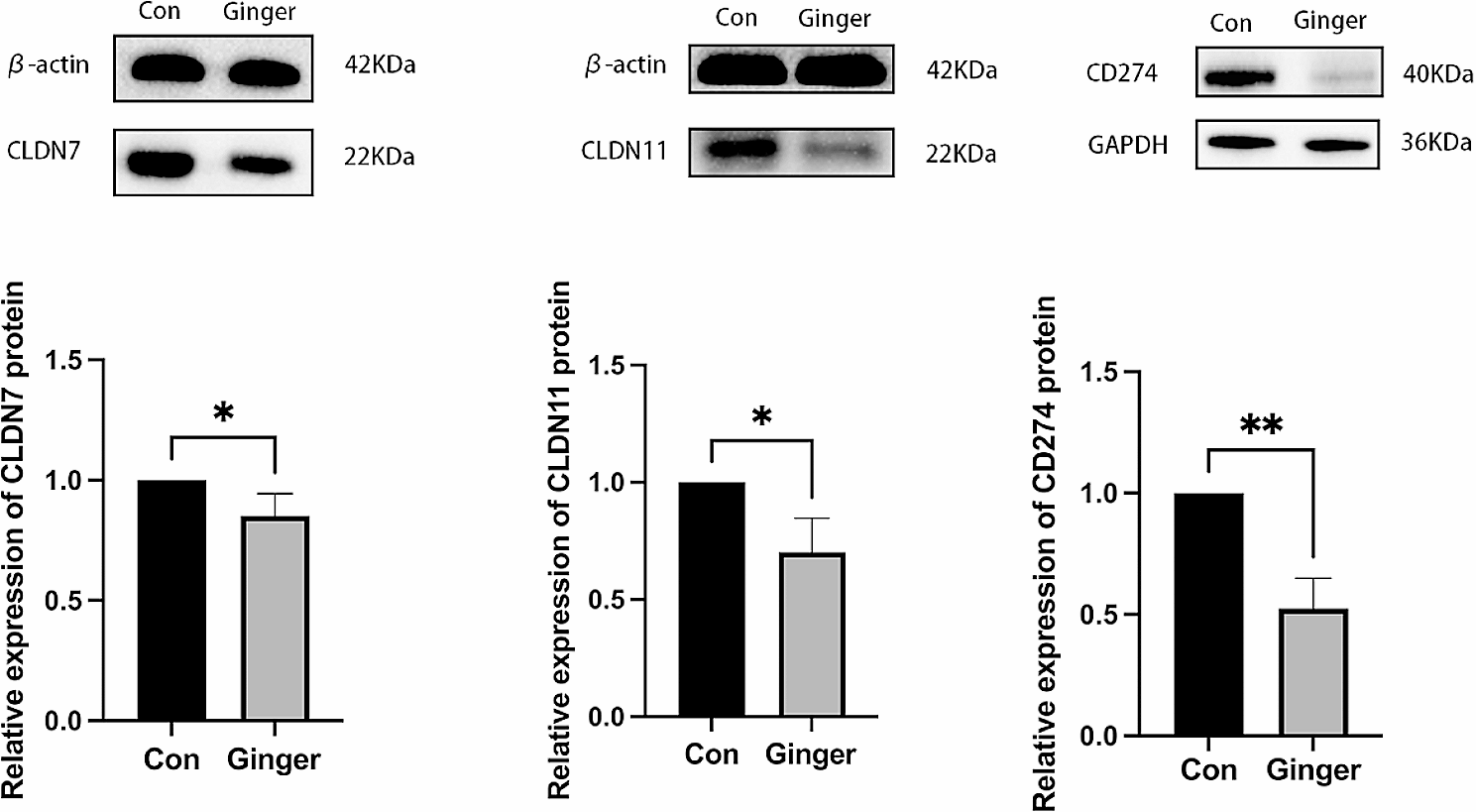
Ginger’s effect on CLDN7, CLDN11, and CD274 mRNA and protein expression. Data are presented as mean ± SEM. *n* = 3. nsp > 0.05, *p < 0.05, **p < 0.01,****p < 0.0001. Con: control group, Ginger: Ginger-treated group

By examining the GO and KEGG pathways, we were able to detect alterations in the m6A methylation of genes associated with cell adhesion molecules (CAMs). These changes were visualized employing the IGV (Fig. 8). The Ginger treatment was reported to affect m6A methylation levels of claudin-7 (CLDN7), claudin 11 (CLDN11), and Programmed Cell Death-Ligand 1(CD274, PD-L1) (Fig. 7). using Western Blot analysis and real-time PCR (RT-PCR), these genes were further analyzed. The outcomes demonstrated that in ovarian cancer cells SKOV3, CLDN7, CLND11, and CD274 were reduced in the Ginger-treated group than in that of the Con group (Fig. 9).

### Effects of Ginger on ovarian cancer cells SKOV3 invasion

**Fig. 10:**
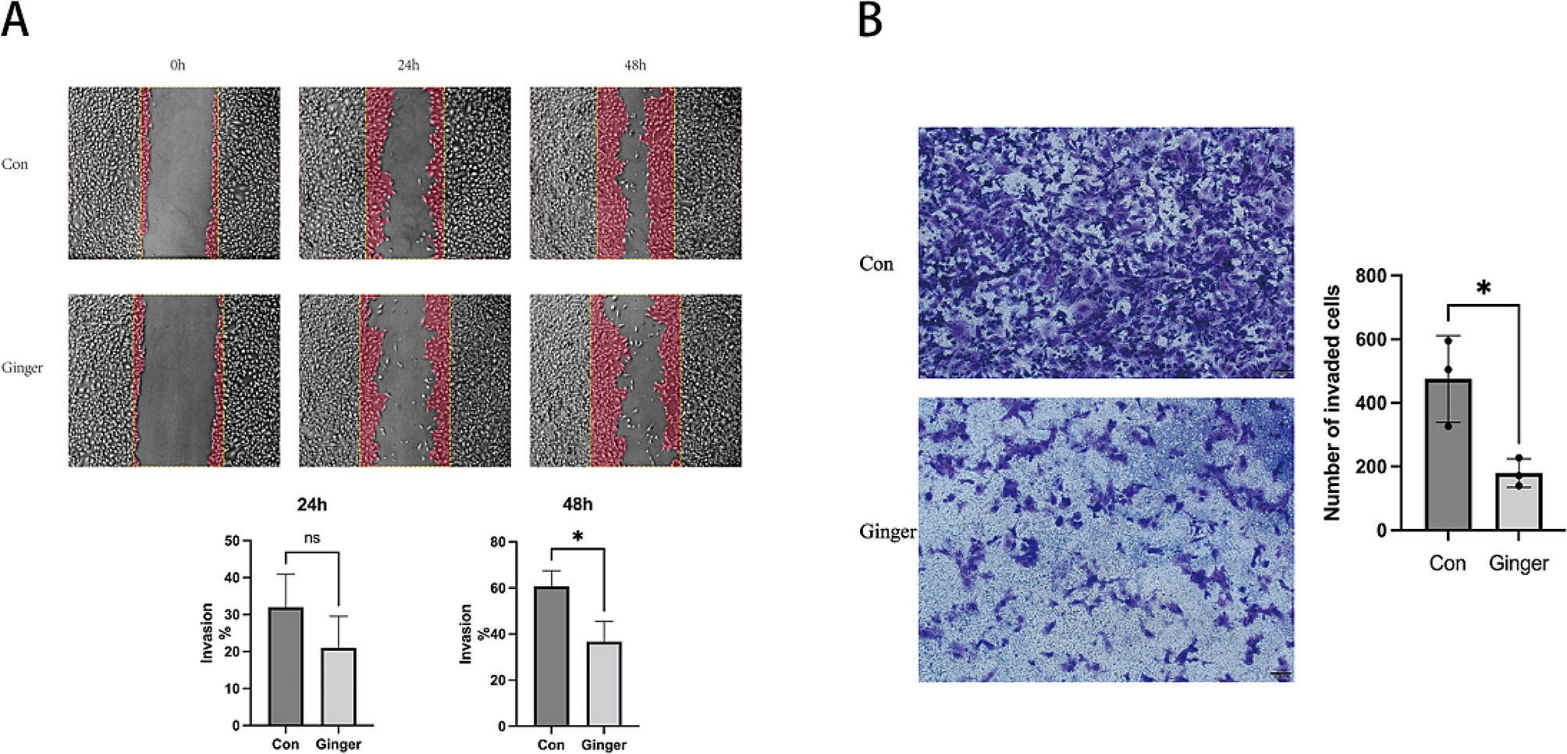
Wound healing assay (**A**) and transwell assays (**B**) (Scale bars = 100 and 200 μm respectively) were applied to investigate ovarian cancer cells’ SKOV3 invasion after the ginger intervention. The data is shown in terms of mean (± SEM, having *n* = 3). ns = *p* > 0.05, *=*p* < 0.05, Con: control group, Ginger: Ginger-treated group

Since CLDN7, CLDN11 and CD274 correlate with the invasive and metastatic ability of tumours, we further did Wound healing assays and transwell assays. The results showed a clear difference in the migration rate of SKOV3 cells between the Con and Ginger-treated groups at the 24 and 48 h treatment time points. In particular, a significant reduction in migration rate was observed in the Ginger-treated group at the 48 h, which was statistically significant (*p* < 0.05). Similarly, the Transwell assay results showed a significant decrease in the migration potential of the Ginger-treated group compared to the Con group at 48 h, as reflected by the cell count (*p* < 0.05). Taken together, these results demonstrate a significant inhibitory effect of ginger on the migratory ability of SKOV3 cells in a time-dependent manner (Fig. 10).

## Materials and methods

### Cell line

SKOV3 cells, acquired from Cell Resource Center, Shanghai Institute of Biology, Chinese Academy of Sciences (CAS, China), were cultured in RPMI 1640 complete medium containing 10% fetal bovine serum. The cells were maintained in a 37 ℃ incubator with 5% CO_2_ and the medium was refreshed every other day, and the cells were passaged once every 2 to 3 days.

### Herbal preparation

The ginger was purchased from the local farmers’ market and identified by Prof Wang Jiafeng of Shandong University of Traditional Chinese Medicine as the fresh rhizome of Zingiber officinale Rosc. Fresh ginger was washed, peeled and cut into slices less than 5 mm thick, soaked in 10 times water for 30 min, decocted for one hour, then soaked in 8 times water and decocted for one hour. Put the decoction in -40℃ for refrigeration, -20℃ for 2 h, put it into freeze dried powder in freeze drying mechanism, and store it at -20℃ for use.

Preparation of drug solution: Take 100 mg ginger freeze-dried powder, dissolve in 10 mL basal medium, filter through 22 μm filter membrane, prepare 10 mg/mL mother liquor for later use (dilute as needed in the experiment).

### Determination of cellular m6A level

The level of m6A modification on the RNA was assessed using the EpiQuickTM Kit (Kit for RNA Methylation; Epigentek Inc. USA). Briefly, a total of 80 µL of binding solution was added to the designated wells, followed by the addition of 2 µL of each of the negative and positive controls (diluted). An RNA sample (200 ng) was also added to the appropriate wells. The samples were then incubated at 37 °C for 90 min. The samples were then incubated with an appropriately diluted capture antibody (50 µL) for 60 min. Enhancer solution and detection antibody (50 µL each, diluted) were then added to the samples. Finally, the samples were incubated with 100 µL of developer solution for 10 min, 100 µL of stop solution was added and the absorbance was recorded (λ = 450 nm).

### Cell migration assay

To assess the migratory capacity of the cells, a wound-healing assay was conducted. The 6-well plates were seeded with a total of 1 × 105 cells, and the ginger-treated group was exposed to ginger for 48 h. When the cells reached 90–100% confluence, a wound was created by manually scraping the cell monolayer with a p200 pipet tip. After 48 h of incubation, images were taken at 0 h, 24 and 48 h using a 100x magnification light microscope (Nikon, Japan). The scratch area at the same location and at different time points was quantified using Image J software (V 1.8.0).

### Cell invasion assay

Cells were inoculated in the upper chamber of Transwell (CorningTM, NY, USA), and after the cells were plastered, equal volumes of normal medium (Con groups) and Ginger medium (Ginger-treated groups) were added respectively. After 48 h of culture and fixed staining, 5 randomly selected fields of view were photographed (100x) and the number of invading cells was counted using Image J software.

### RNA extraction and real-time quantitative PCR

Total RNA was extracted from SKOV3 cells using the SPARK easy cell RNA kit (Sparkjade, Shandong, China) according to the manufacturer’s instructions. Subsequently, 2 mg of the total RNA was utilized for the synthesis of first-strand cDNA employing HiScript III RT SuperMix (Vazyme, Nanjing, China). The resulting cDNA served as the template for quantitative real-time PCR (qRT-PCR). The mRNA expression levels were assessed employing the ChamQ Universal SYBR qPCR Master Mix (Q711, Vazyme, China). To ensure accurate normalization, gene expression was standardized to β-actin expression. Data analysis was performed utilizing the 2 − ΔΔCt protocol. The sequences of primers employed in this research are provided in Table 2.

### Western blot analysis

Protein extraction from Cells was executed by employing RIPA lysis buffer, accompanied by diverse treatments with protease inhibitors (Solarbio, Beijing, China). The quantification of the protein concentration was carried out utilizing the BCA protein detection kit (Solarbio, Beijing, China). To achieve protein separation, SDS-PAGE (Solarbio, Beijing, China) was employed, followed by the transfer of the proteins to PVDF membranes using an electroblotting apparatus (Millipore, Burlington, USA). Subsequently, the membranes were left to incubate for an overnight duration at 4 °C with primary antibodies of KIAA1429(1/1000, Abcam), FTO (1/1000, Bioss), CLDN7(1/1000, Abclonal), CLDN11 (1/1000, Abclonal), CD274 (1/1000, Proteintech), GAPDH (1/1000, CST), and β-actin (1/5000, Abcam). Post being washed thrice with 0.1% TBST, the membranes were left to incubate with a secondary antibody (1:1,000; Cell Signaling Technology, Danvers, MA, USA) at room temperature for 2 h. The bands were visualized utilizing an enhanced chemiluminescence (ECL) detection reagent (Millipore, Burlington, MA, USA). Finally, the quantification of band intensity was conducted employing the Image J software.

### Methylated RNA immunoprecipitation and sequencing (m6A MeRIP-Seq)

Extract: Total RNA extraction and purification were conducted through the utilization of TRIzol reagent (Invitrogen, Carlsbad, USA) as per the manufacturer’s protocol. The RNA quantity and purity of all the samples were quantified through NanoDrop ND-1000 (NanoDrop, Wilmington, USA). RNA integrity was determined employing an Agilent Bioanalyzer 2100 (Agilent, USA) having an RIN value greater than 7.0, and authenticated through electrophoresis with agarose denaturing gel. Poly(A) RNA was extracted from 50 µg total RNA twice utilizing Dynabeads Oligo (dT)25-61005 (Thermo Fisher, USA) to improve its purity.

Construction of Library: The poly(A) RNA (purified) was fractionated employing a Magnesium RNA Fragmentation Module (cat No. e6150; NEB; USA) at a temperature of 86 ℃ for 7 min. Subsequently, the cleaved RNA fragments were incubated with an m6A-specific antibody (cat No. 202,003; Synaptic Systems; Germany) in an IP (Immunoprecipitation) buffer (containing 750 mM NaCl, 50 mM Tris-HCl, and 0.5% Igepal CA-630) at 4 ℃ for 2 h. Thereafter, reverse-transcription of IP RNA into complementary DNA (cDNA) was performed utilizing SuperScript™ II Reverse Transcriptase (Invitrogen, cat. 1,896,649, USA). The generated cDNA was then utilized to construct U-labeled second-stranded DNAs using DNA polymerase I (E. coli; cat no. m0209; NEB; USA), RNase H (cat no. m0297; NEB; USA), and dUTP Solution (cat no. R0133; Thermo Fisher; USA). To facilitate ligation to the indexed adapters, the blunt ends of each strand were provided with an A-base. Each adapter included a T-base overhang, allowing for the ligation of the adapter to the fragmented DNA (A-tailed). Subsequently, the A-tailed DNA was combined with single- or dual-index adapters, and the desired size range was carefully chosen using AMPureXP beads. The ligated products were subjected to PCR amplification using the following conditions: an initial denaturation for 3 min at 95 ℃, followed by 8 denaturation cycles for 15 s at 98 ℃, annealing at 60 ℃ for 15 s, and extension at 72 ℃ for 30 s. The final extension step was performed at 72℃ for 5 min. The resulting cDNA library had an average size of 300 ± 50 bp. Paired-end sequencing (PE150) with 2 × 150 bp read length was carried out using the Illumina Novaseq™ 6000, following the recommended protocol provided by the vendor.

### Statistical analysis

The data were presented as mean ± SEM and were analyzed utilizing the t-test and one-way analysis of variance (ANOVA) in SPSS Statistics v20.0 software. Statistical significance was determined using *P* values less than 0.05.

**Table 2:** The primers sequences used in this study

RNAs	Primer-F(5’-3’)	Primer-R(5’-3’)
**GAPDH**	GCACCGTCAAGGCTGAGAAC	TGGTGAAGACGCCAGTGGA
**WTAP**	GCAACAACAGCAGGAGTCTG	TCGCTGGGTCTACCATTGTT
**HNRNPA2B1**	AGACTGTGTGGTAATGAGGGA	GCTACAGCACGTTTTGGCTC
**YTHDC1**	AGGAAAGTCAGCCACAGAGT	GCGTAGGAGATTTGGCCCTC
**YTHDF2**	AGCCCCACTTCCTACCAGATG	TGAGAACTGTTATTTCCCCATGC
**YTHDF1**	CATCTTCGACGACTTTGCTCACTA	ACTGGAGCTGACCAAGCACAC
**IGF2BP1**	CGTCTCATTGGCAAGGAAGGA	CTCTCAGGGTTGTAAAGGGTAAGG
**METTL3**	CTGTCGCAAGCTGCACTTCA	CTCAGAATCCATGCAAGCATCA
**ALKBH5**	TGTGCTTCGGCTGCAAGTTC	CCTGAGGCCGTATGCAGTGA
**KIAA1429**	CGACGAACAGTAGACAGTATTCCT	AGTCAGCAATTCCATCTTCATCAC
**FTO**	TGCAGAATGTCTGTGACGATGTG	TTTCTGTATCGATTGCCTTGAAACC
**CLDN11**	TGACTGCCTGCTTTGTGCT	CCGCCTGTACAAGCGAATTAC
**CD274**	TGCCGACTACAAGCGAATTAC	GAATTGGTGGTGGTGGTCTTAC
**CLDN7**	GGGAGACGACAAAGTGAAGAAGG	TGTTGGTAGGGATCAAAGGGTTAT

## Discussion

The challenge in the management of ovarian cancer is the lack of early screening and diagnostic methods, which means that it is often detected at an advanced stage. Moreover, ovarian cancer is prone to recurrence and drug resistance following surgery and radiotherapy [21]. In recent years, numerous studies have identified the potential of botanical drugs with anti-tumour activity [22]. Ginger, native to China and South East Asia, is used as a common spice in many places and many people believe that eating more ginger has anti-cancer properties [23, 24]. It is also well documented that ginger and its active compounds can exert anti-tumour effects through their modulation of oncogenes, cell cycle, apoptosis, transcription factors, angiogenesis, and growth factors, ultimately inhibiting the growth of a variety of tumours [25, 26]. In our previous study, we observed a variety of ovarian cancer cells (HOSEPIC, SKOV3, OVCAR3, A2780, etc.) and found that ginger had the most obvious inhibitory effect on the proliferation of SKOV3 cells (see Supplementary Fig. 1). Furthermore, the current study revealed a significant inhibition of proliferation and migration in SKOV3 ovarian cancer cells following treatment with ginger.

However, the mechanisms underlying the anti-tumour proliferative and anti-metastatic effects of ginger are unclear. We hypothesize that ginger, as a common spice, affects tumourigenesis, development and metastasis through epigenetic alterations. By influencing the degree of m6A methylation alterations, which in turn have a significant impact on tumor cell proliferation, invasion, and metastasis as well as treatment resistance, m6A-associated regulatory proteins have an essential function in regulating the growth of malignant tumors [27, 28]. The m6A-associated regulatory proteins play an important role in tumour cell proliferation, invasion and metastasis, and drug resistance [29, 30]. It has been shown that phytopharmaceuticals can influence the level of m6A methylation modifications to regulate disease development and progression by affecting m6A. We found that ginger could significantly reduce the overall m6A level in ovarian cancer cells SKOV3. Further RT-PCR detected m6A methylation-related genes and found that the levels of FTO, KIAA1429, YTHDC1, YTHDF1 and YTHDF2 mRNA were significantly changed in the ginger-treated group, and we corroborated the protein expression results of FTO and KIAA1429 with Western Blot to be consistent with the gene expression results.

We use Kaplan-Meier - Plotter database analysis (http://www.kmplot.com/analysis/), the result shows: High expression of ALKBH5, HNRNPA2B1, IGF2BP1, KIAA1429, YTHDC1, METTL3, YTHDF1 and YTHDF2 was associated with shorter progression-free survival (PFS) in ovarian cancer patients. However, patients with high expression of FTO and WTAP tended to have longer PFS. (see Supplementary Fig. 2) These results indicate that ginger can affect the m6A modification level of some mrnas by regulating multiple m6A modification regulators, and then affect the proliferation, invasion, drug resistance and other biological processes of tumors.

We further sequenced m6A methylation in the Con groups and Ginger-treated groups. Analysis of the m6A methylation combined with RNA expression sequencing results revealed that ginger could affect multiple targets and pathways through m6A modifications. Therefore, altering the m6A methylation levels of CLDN7, CLDN11, and CD274, which in turn affects the growth of SKOV3 in ovarian cancer cells may be one of the mechanisms by which ginger resists ovarian cancer cells SKOV3.

Cell adhesion molecules (CAMs) are major cytokines that mediate cell attachment and have a significant function in cell-cell and cell-extracellular matrix adhesion [31]. Aravindakshan et al [32] found overexpression of claudin-11 in mouse ovarian plasmacytic cystadenoma. CD274 (PD-L1), which is abundantly expressed in many tumours, induces apoptosis in CTL and helps tumour cells to evade immune surveillance by activating CD274 receptors on CTL (cytotoxic T lymphocytes), thereby increasing drug resistance in tumour cells and making them more difficult to treat [33]. In line with the sequencing data, our investigation discovered that ginger inhibited the expression of CLDN7, CLDN11, and CD274 in ovarian cancer cells SKOV3. Therefore, by altering the m6A methylation levels of CLDN7, CLDN11, and CD274, which in turn affects the multiplication of ovarian cancer cells SKOV3 may be one of the mechanisms by which ginger is anti-SKOV3.

The multi-component and multi-target characteristics of Chinese medicine are one of the characteristics of Chinese medicine treatment, but they also add difficulty to the related research. The results of this experiment showed that ginger not only adjusted m6A methylation enzyme FTO, demethylase KIAA1429 affected m6A levels in tumour cells, but also had significant effects on the expression of various readers, but the specific components involved in this process and their specific mechanisms need to be further investigated, and how the expression of these m6A modification-related proteins interacted with the modification of related mRNAs also needs to be further investigated.

### Electronic supplementary material

Below is the link to the electronic supplementary material.

Supplementary Material 1

Supplementary Material 2

Supplementary Material 3

Supplementary Material 4

Supplementary Material 5

Supplementary Material 6

Supplementary Material 7

**Supplementary Material 8: Fig S1** The IC50 values of four ovarian cancer cells treated with ginger at 24 h.

**Supplementary Material 9: Fig S2** Kaplan Meier survival curves of m6A modifier regulator expression in ovarian cancer.

## Data Availability

The datasets generated and/or analysed during the current study are available in the [NCBI] repository, reference number [PRJNA1033812].
