# Supplementary figures and images for: Ginger inhibits the invasion of ovarian cancer cells SKOV3 through CLDN7, CLDN11 and CD274 m6A methylation modifications
Source: BMC Complement Med Ther. 2024 Apr 4;24:145. doi: 10.1186/s12906-024-04431-3 (PMC10993429; doi:10.1186/s12906-024-04431-3)

QC01

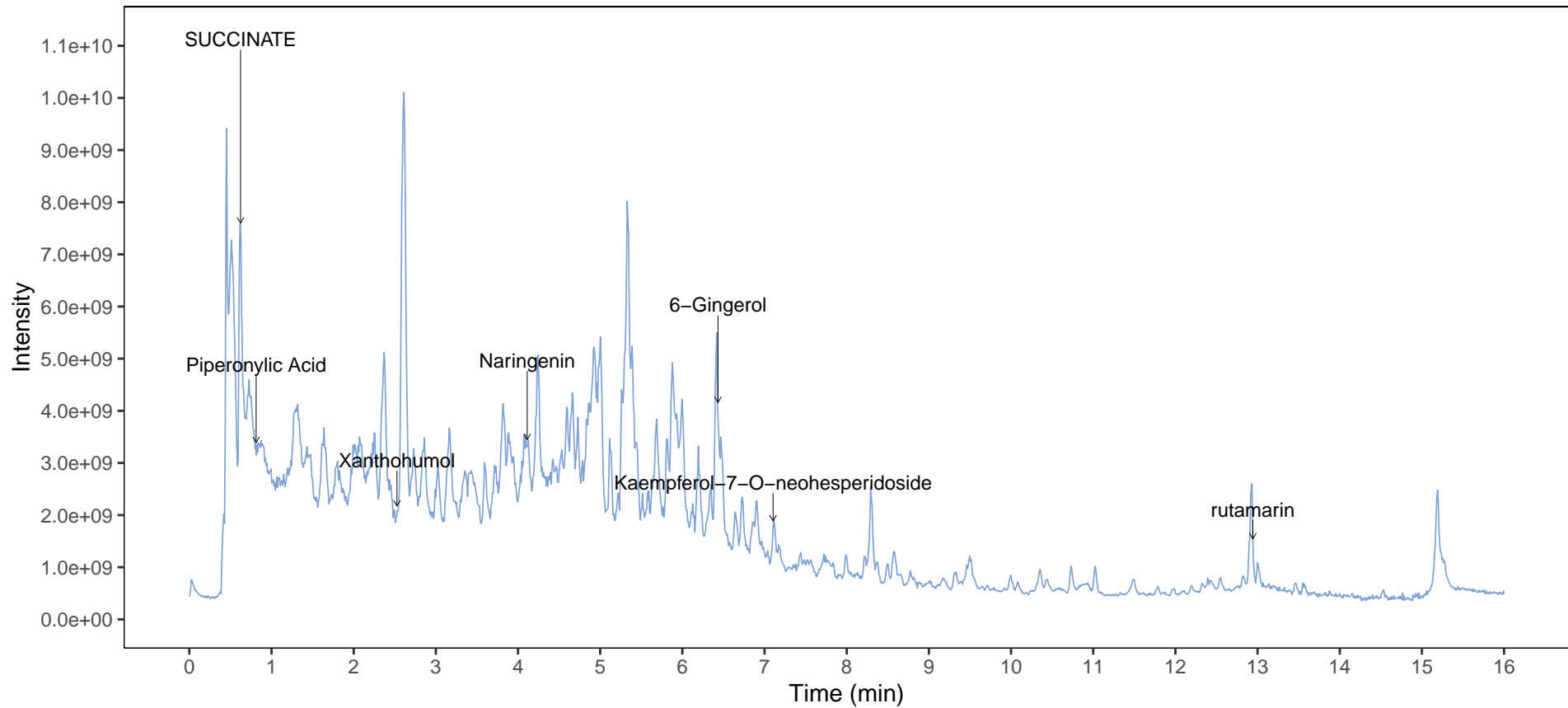

Supplement: Supplementary file 1 — Supplementary Material 1 [file 12906_2024_4431_MOESM1_ESM.pdf]

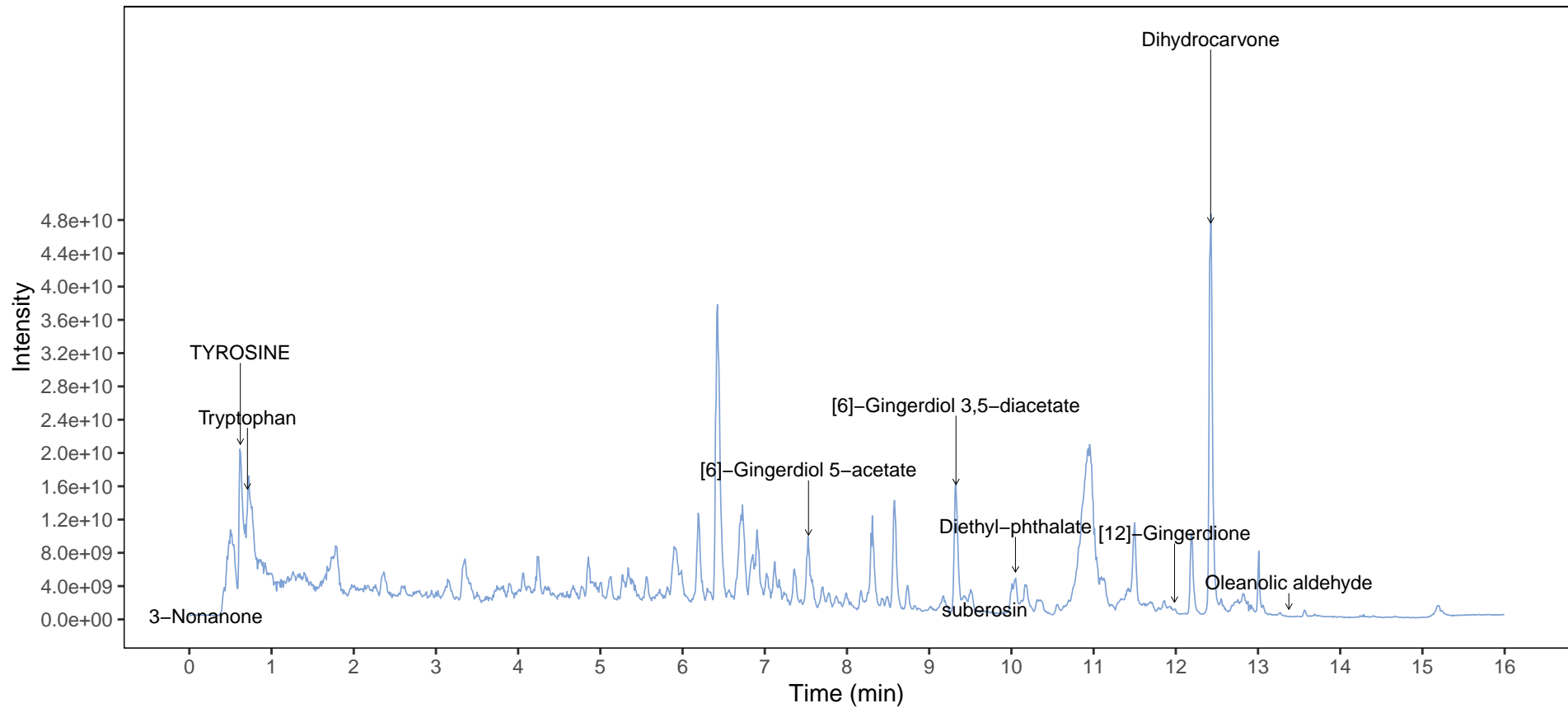

Supplement: Supplementary file 2 — Supplementary Material 2 [file 12906_2024_4431_MOESM2_ESM.pdf]

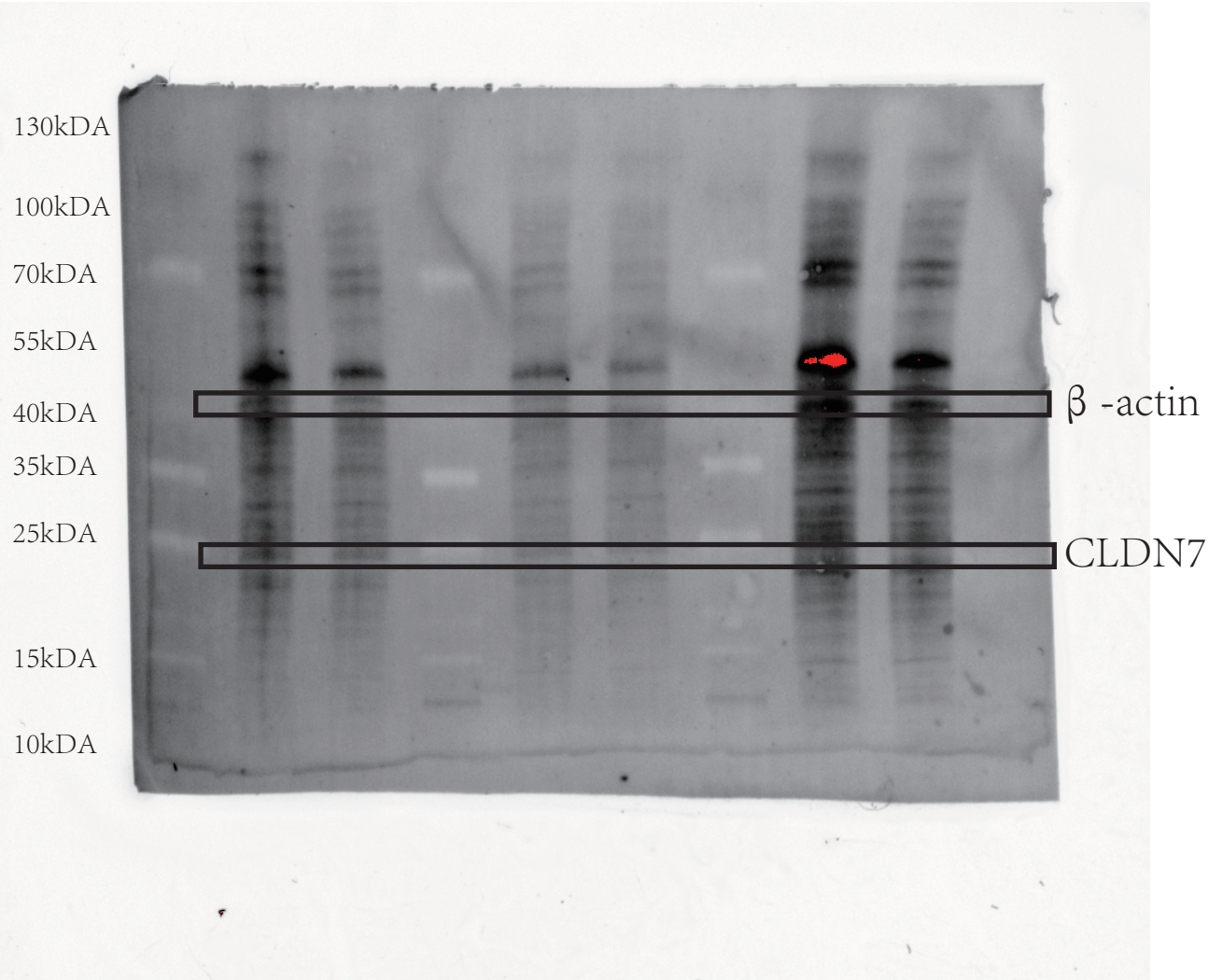

PVDF membrane

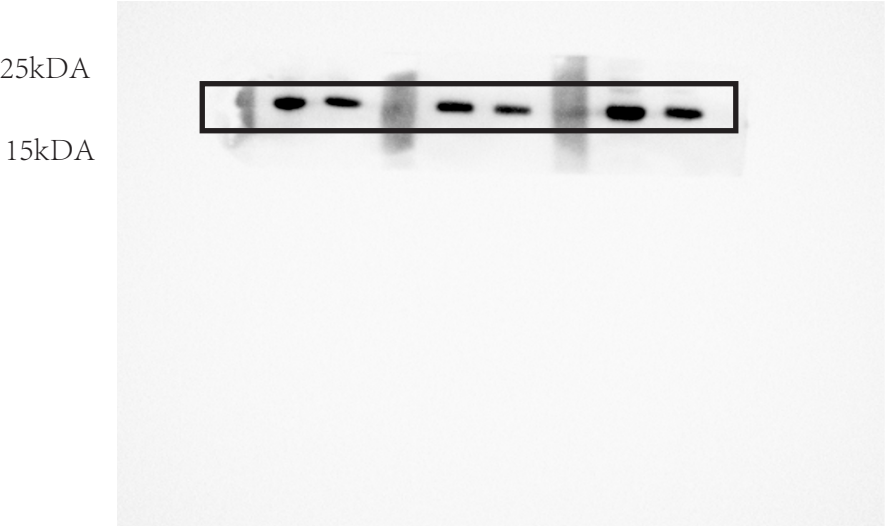

CLDN7

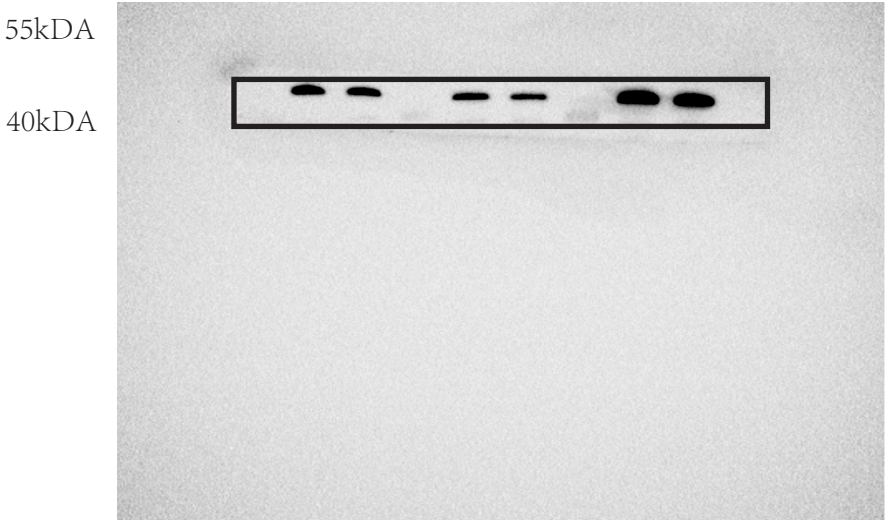

$\beta$ -actin

Supplement: Supplementary file 3 — Supplementary Material 3 [file 12906_2024_4431_MOESM3_ESM.pdf]

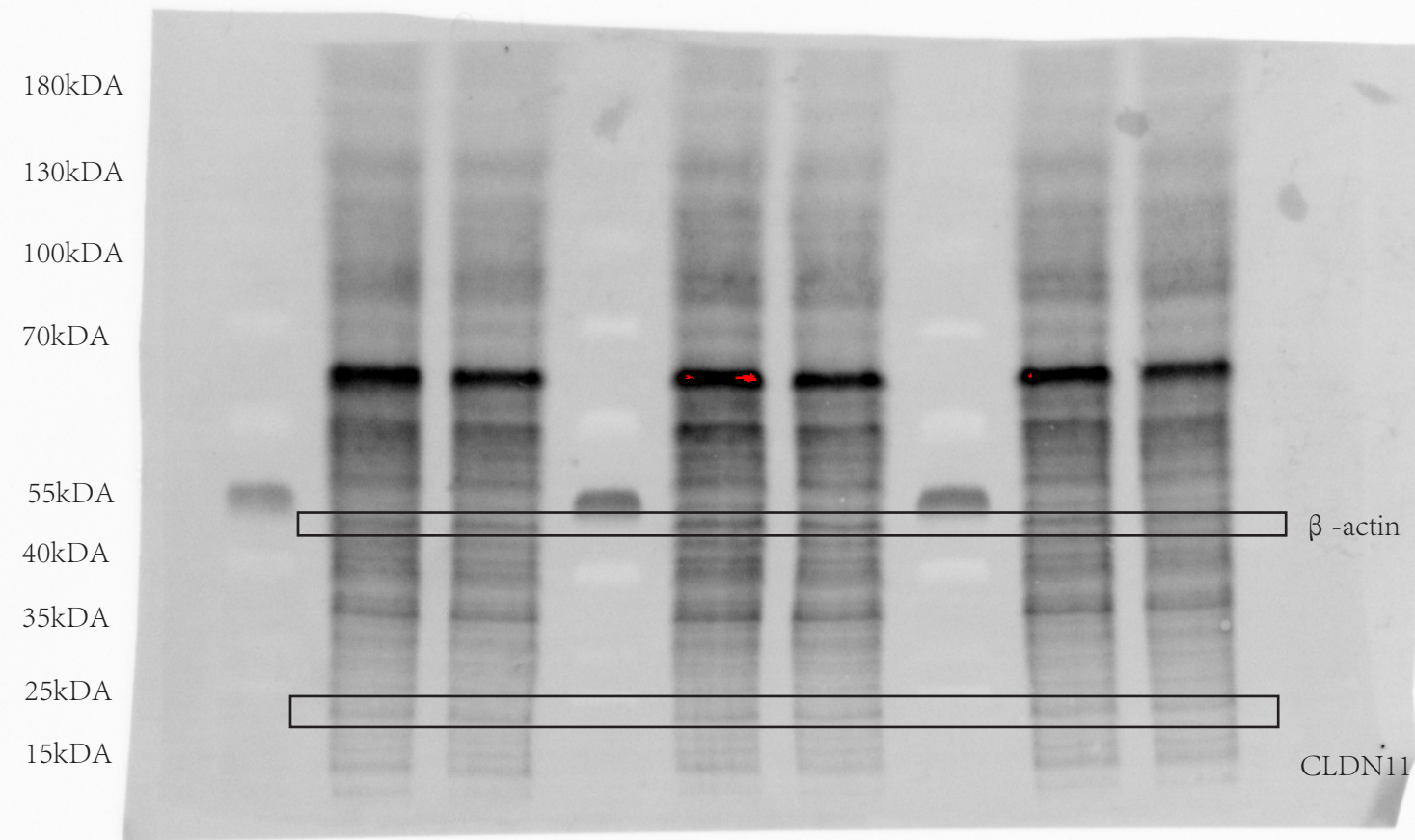

PVDF membrane

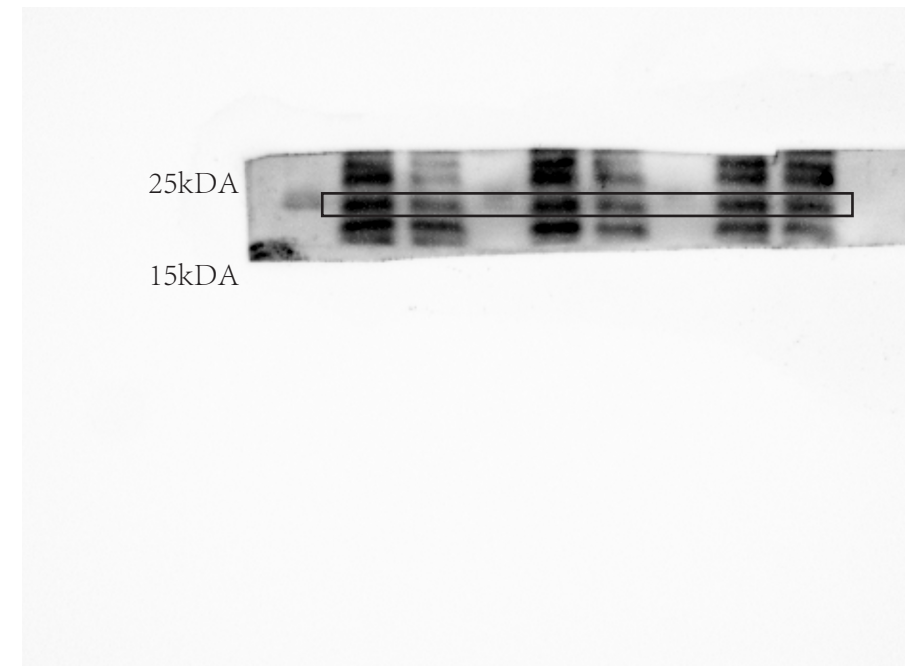

CLDN11

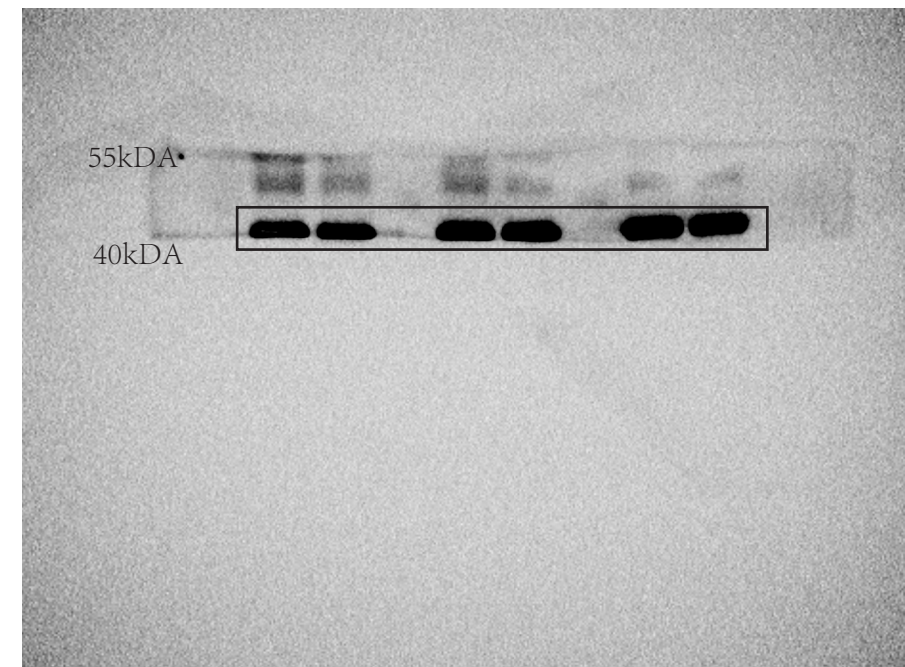

$\beta$ -actin

Supplement: Supplementary file 4 — Supplementary Material 4 [file 12906_2024_4431_MOESM4_ESM.pdf]

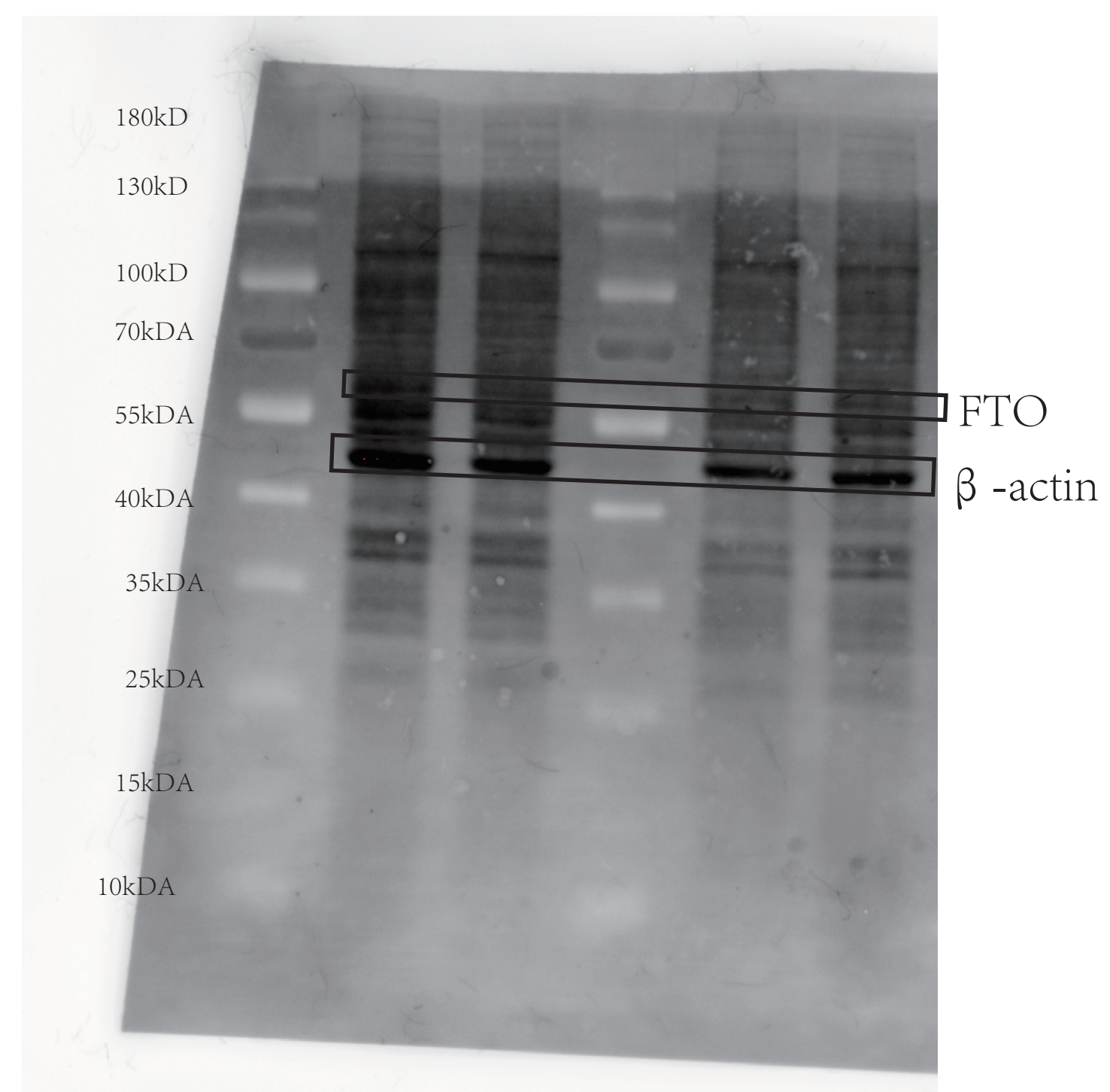

PVDE membrane

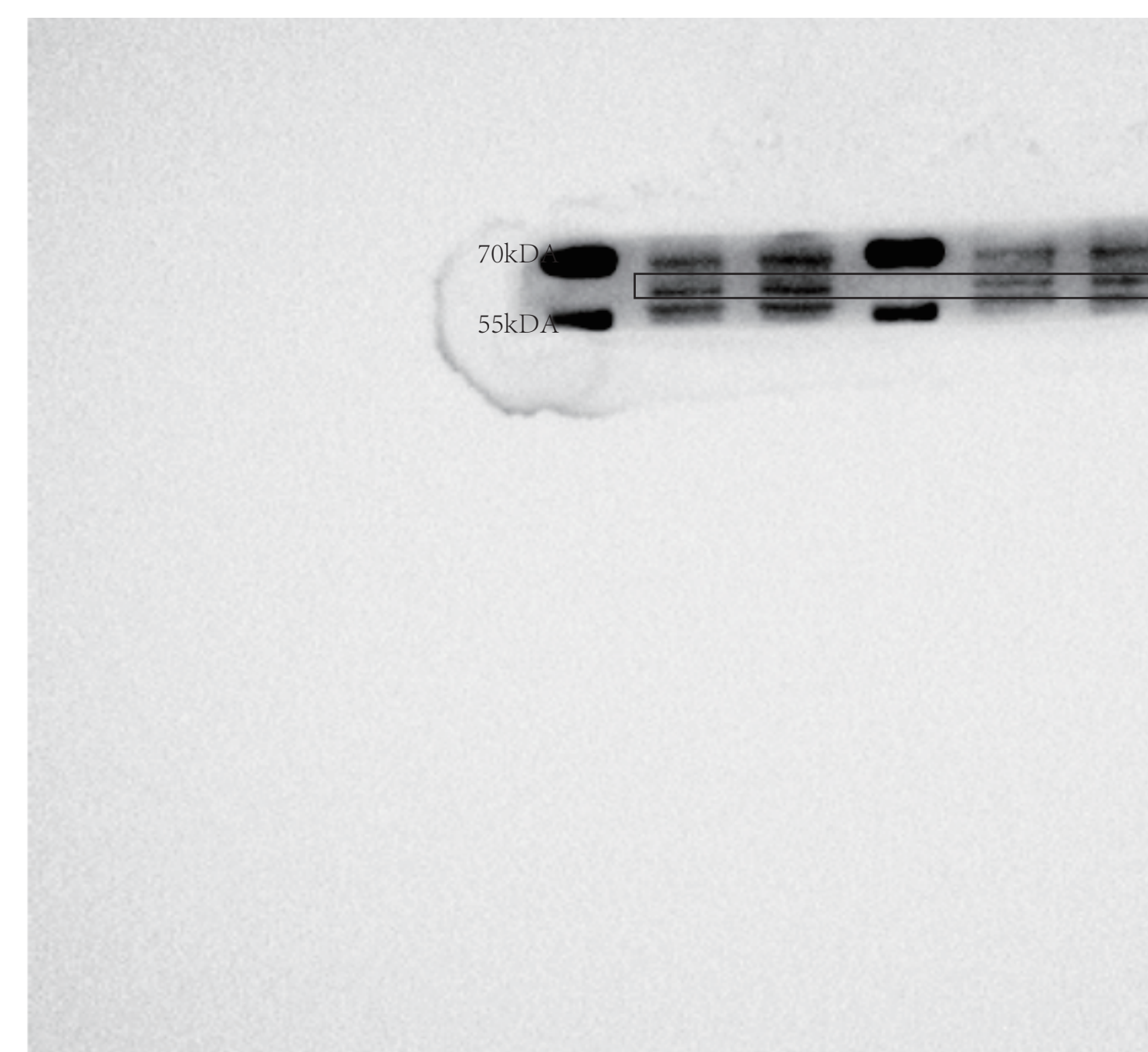

FTO

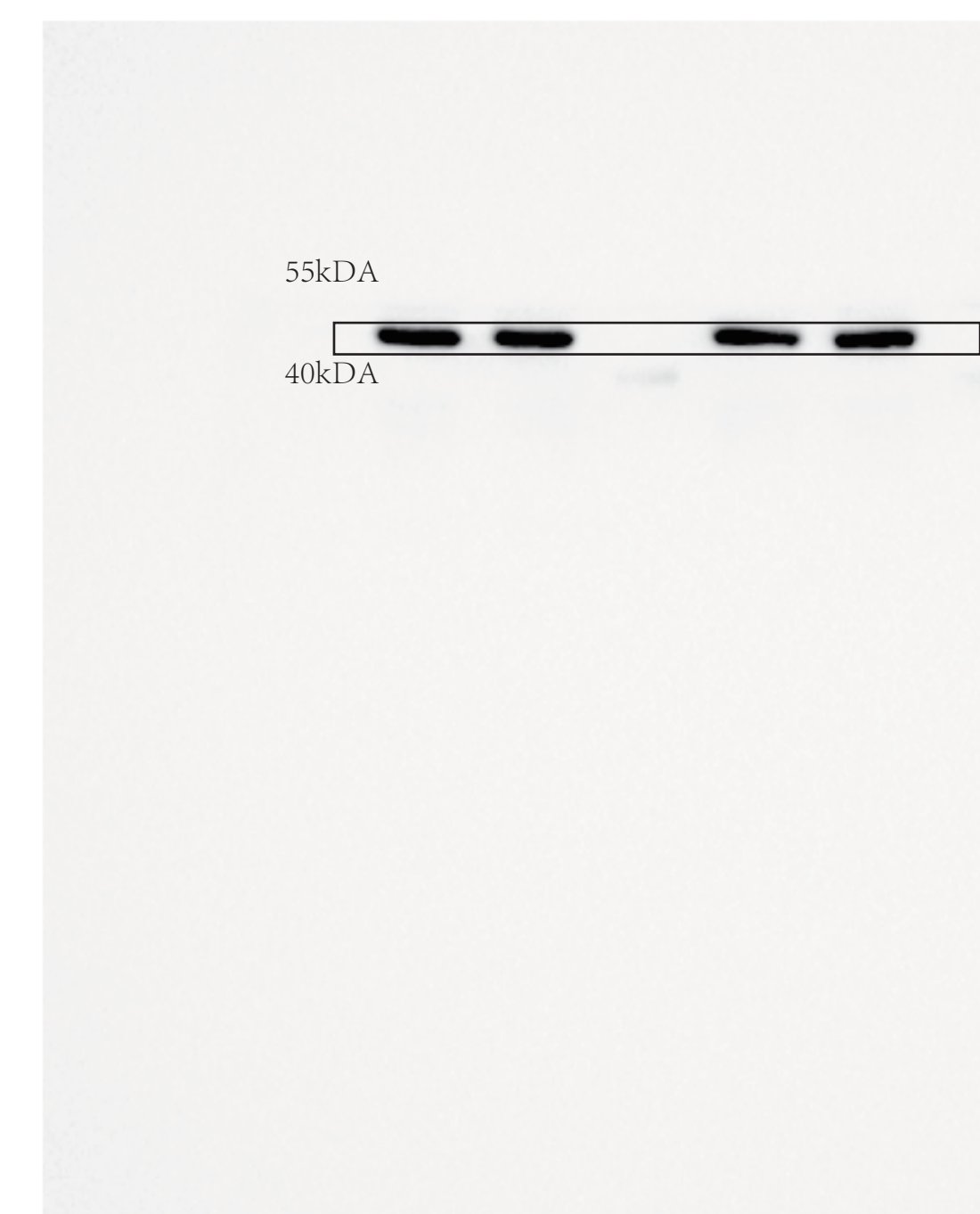

$\beta$ -actin

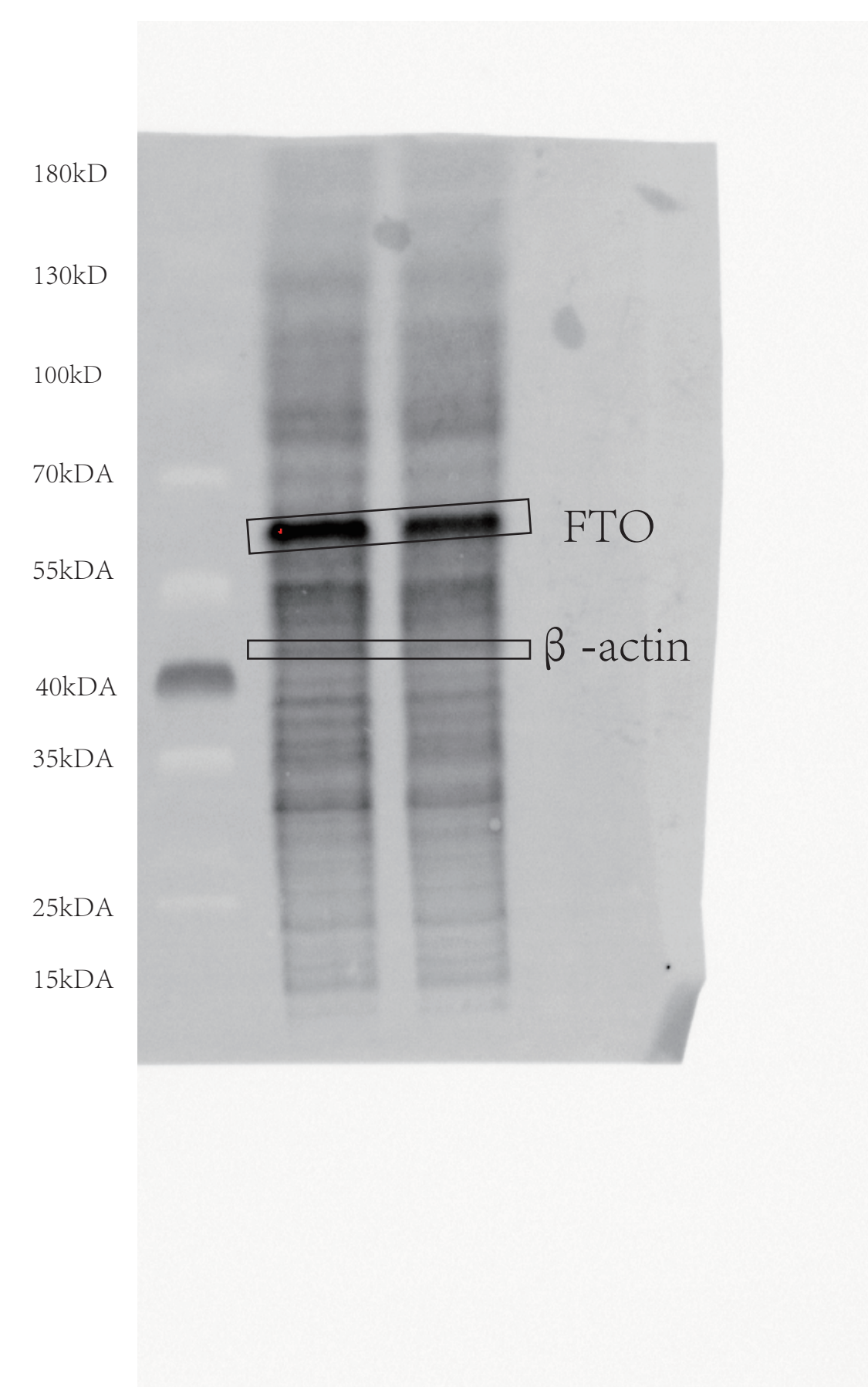

PVDE membrane

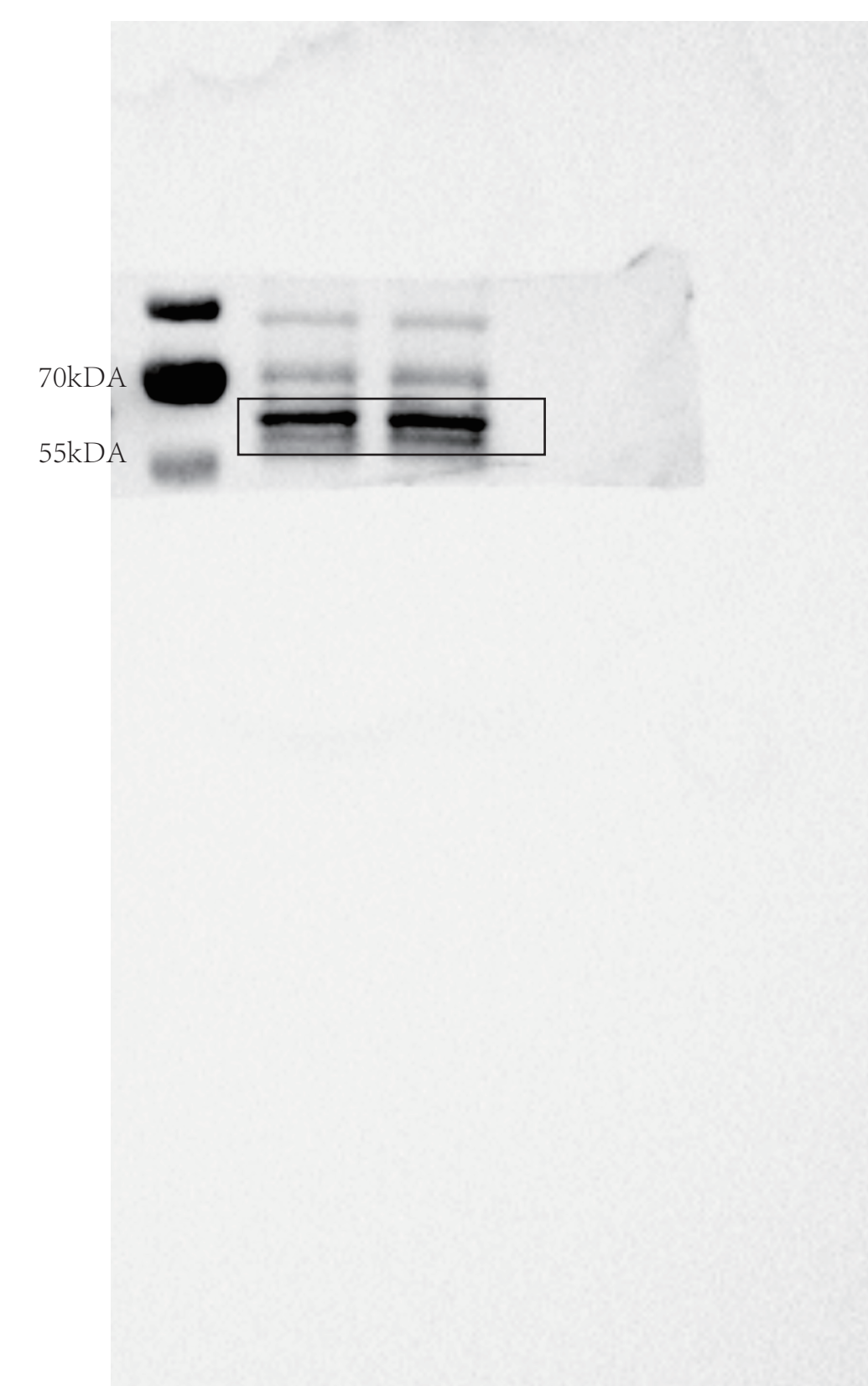

FTO

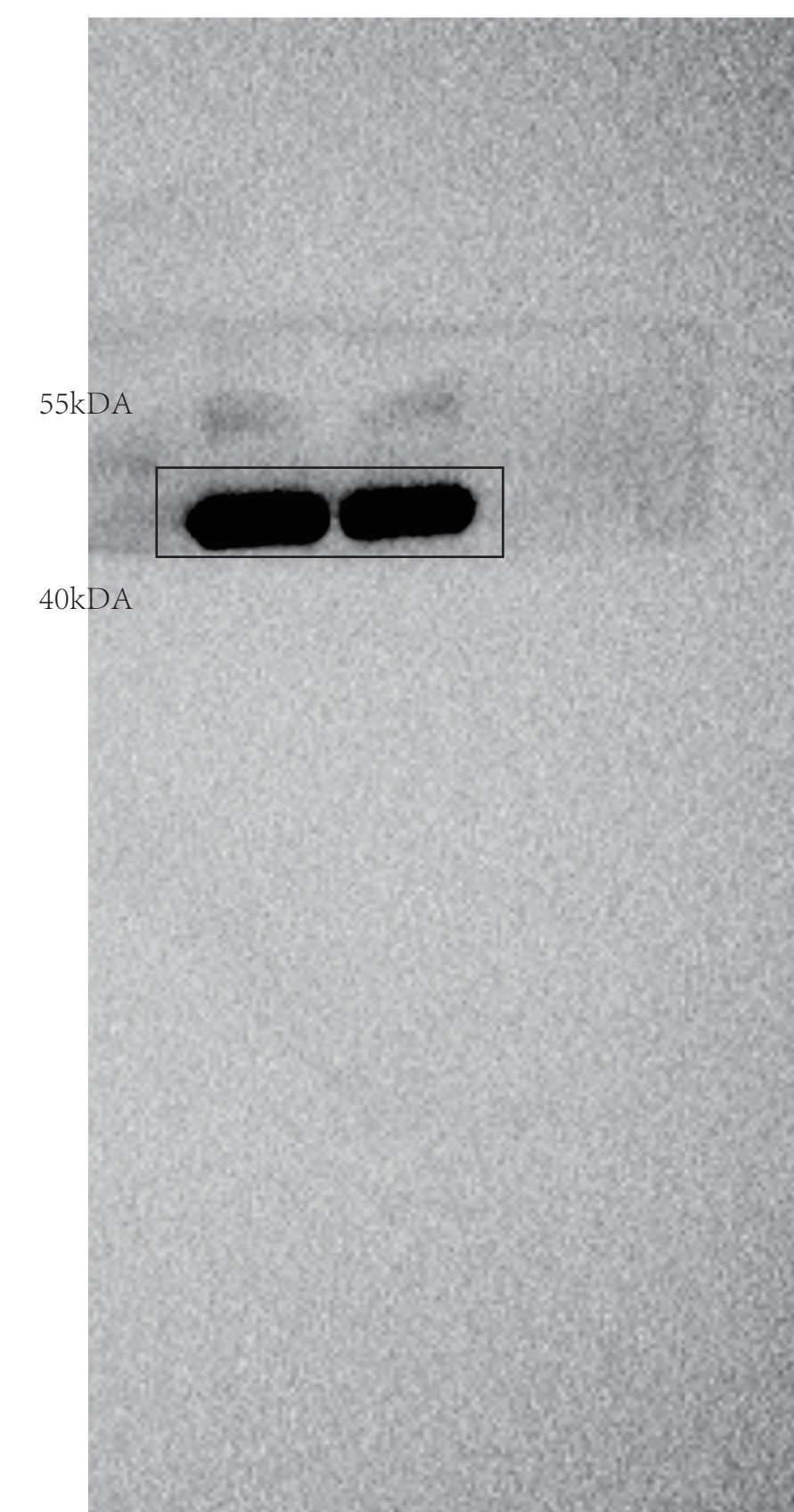

$\beta$ -actin

Supplement: Supplementary file 5 — Supplementary Material 5 [file 12906_2024_4431_MOESM5_ESM.pdf]

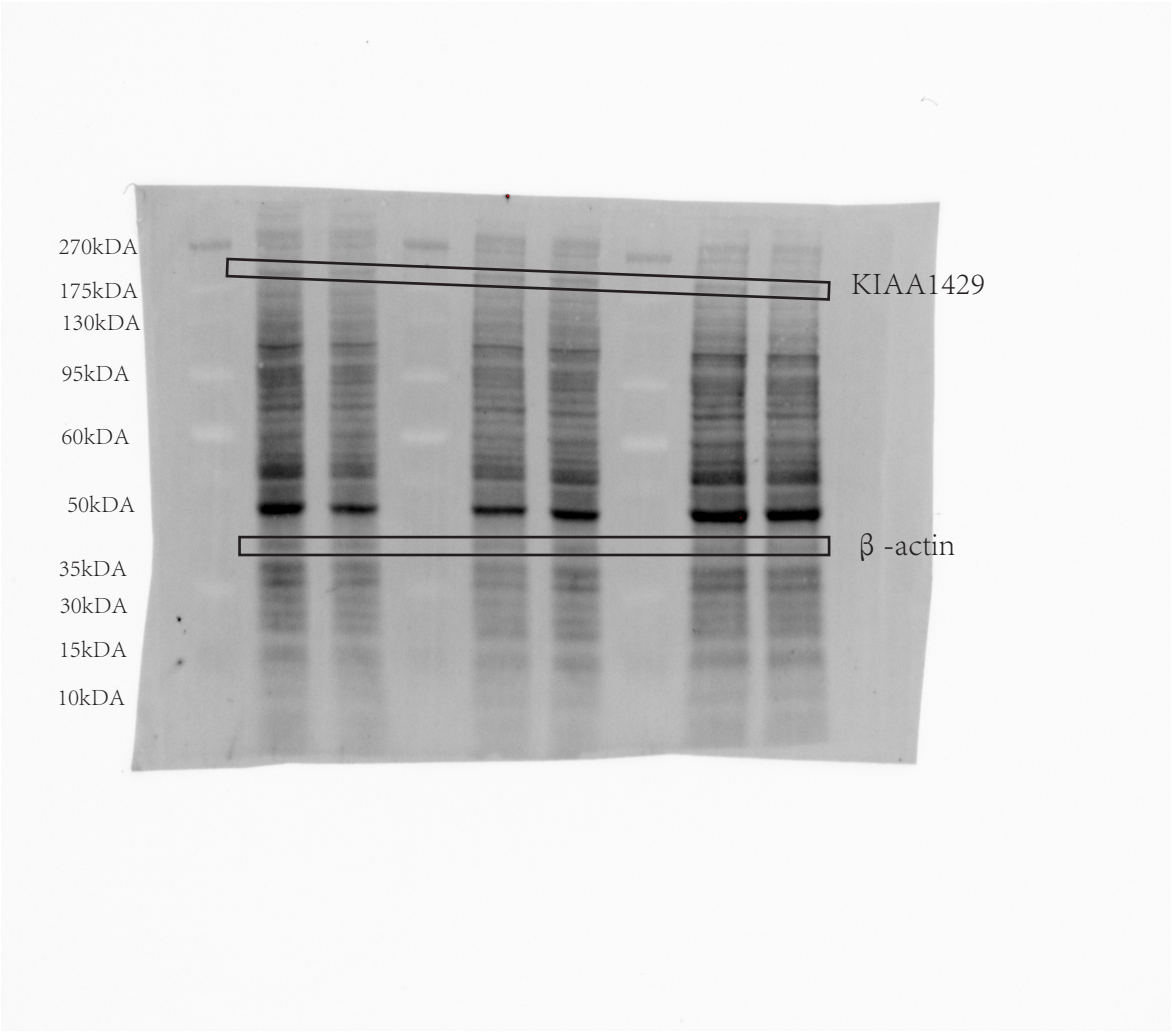

PVDF membrane

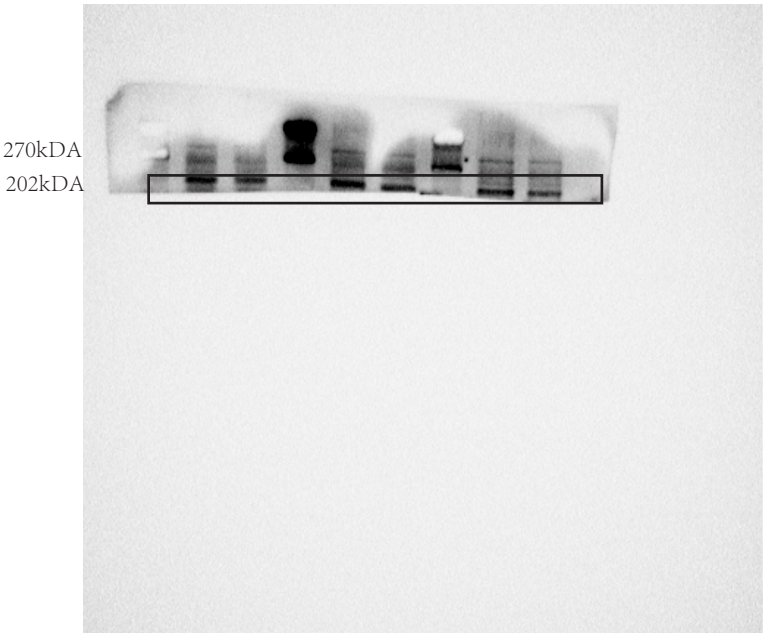

KIAA1429

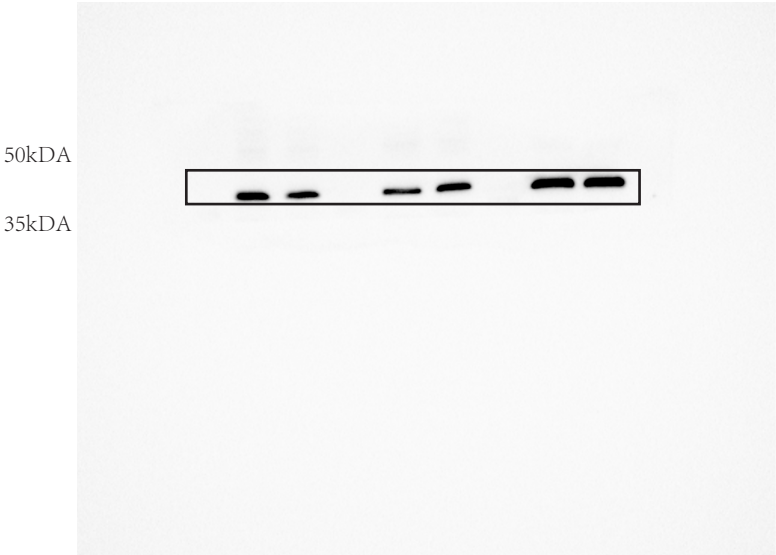

$\beta$ -actin

Supplement: Supplementary file 6 — Supplementary Material 6 [file 12906_2024_4431_MOESM6_ESM.pdf]

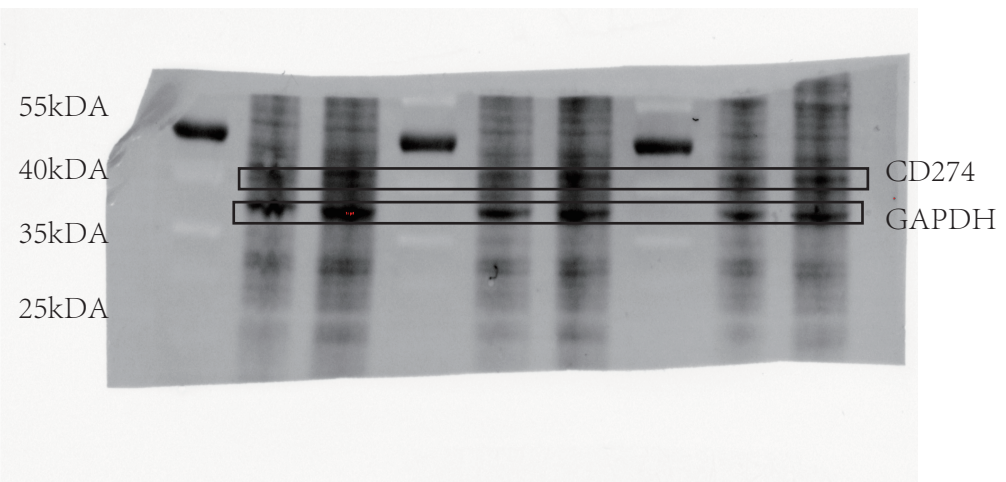

PVDF

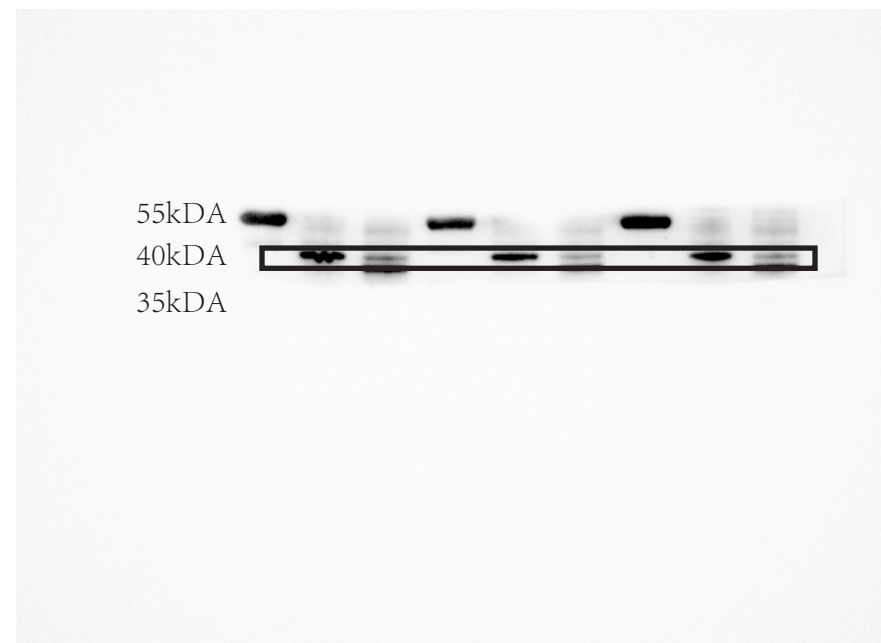

CD274

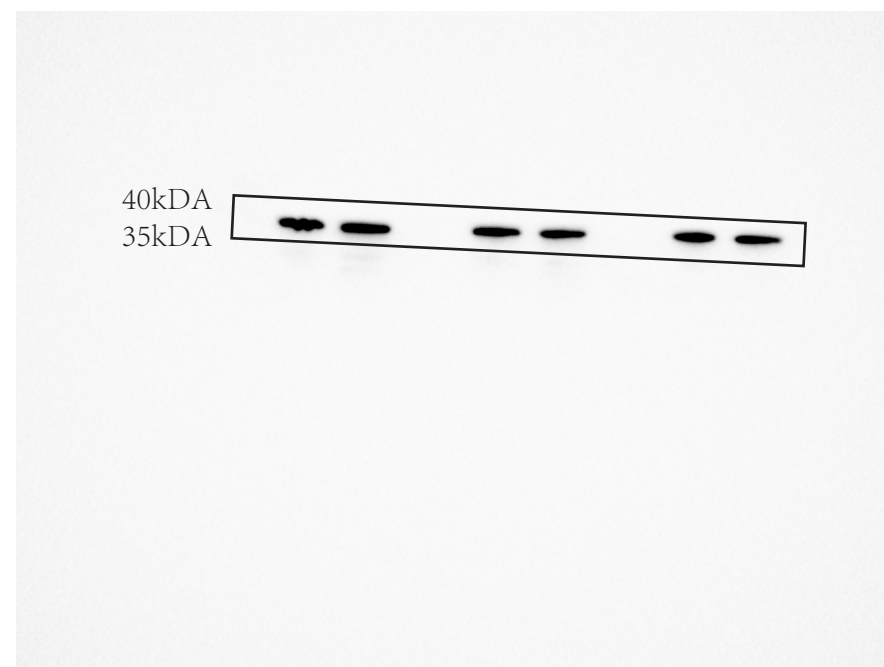

GAPDH

Supplement: Supplementary file 7 — Supplementary Material 7 [file 12906_2024_4431_MOESM7_ESM.pdf]

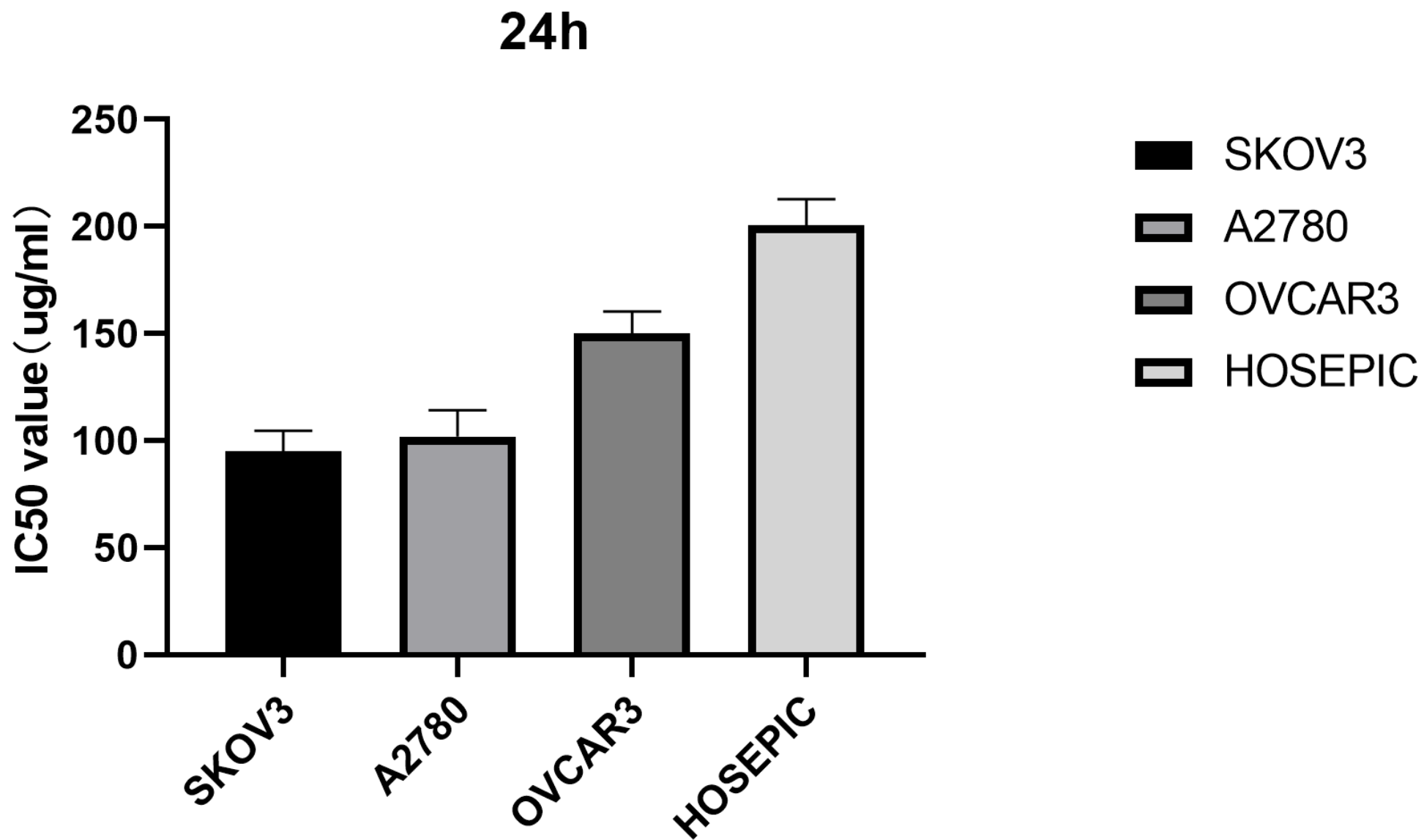

Fig S1 The IC50 values of four ovarian cancer cells treated with ginger at 24h

Supplement: Supplementary file 8 — Supplementary Material 8: Fig S1 The IC50 values of four ovarian cancer cells treated with ginger at 24 h. [file 12906_2024_4431_MOESM8_ESM.pdf]

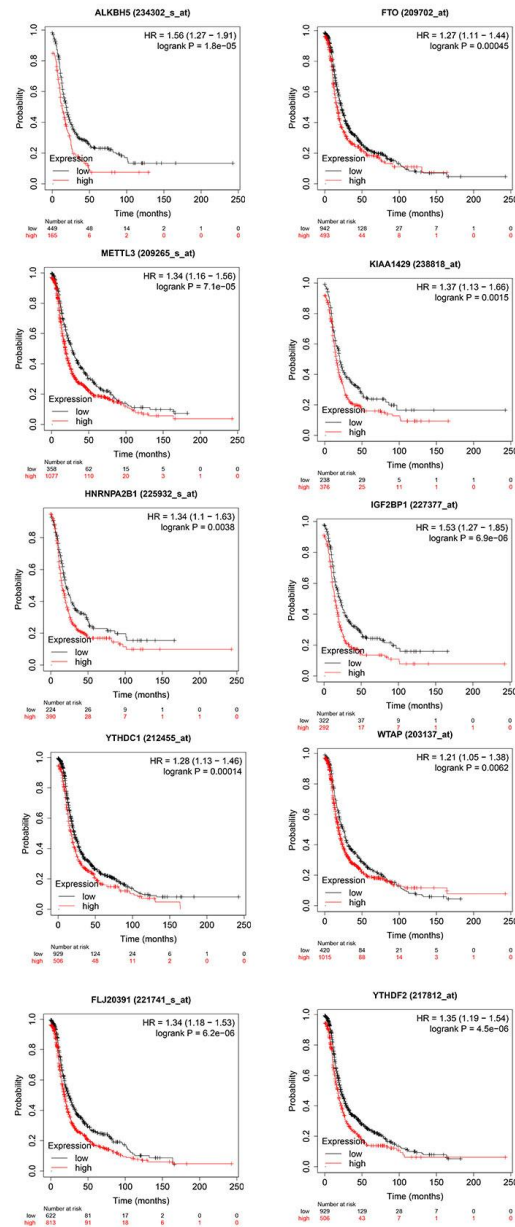

Fig S2 Kaplan Meier survival curves of m6A modifier regulator expression in ovarian cancer

Supplement: Supplementary file 9 — Supplementary Material 9: Fig S2 Kaplan Meier survival curves of m6A modifier regulator expression in ovarian cancer. [file 12906_2024_4431_MOESM9_ESM.pdf]
